# Epigenetic Patterns Maintained in Early *Caenorhabditis elegans* Embryos Can Be Established by Gene Activity in the Parental Germ Cells

**DOI:** 10.1371/journal.pgen.1001391

**Published:** 2011-06-09

**Authors:** Jackelyn K. Arico, David J. Katz, Johan van der Vlag, William G. Kelly

**Affiliations:** 1 Biology Department, Rollins Research Center, Emory University, Atlanta, Georgia, United States of America; 2 Graduate Program in Biochemistry and Cell and Developmental Biology, Emory University, Atlanta, Georgia, United States of America; 3 Nephrology Research Laboratory, Nijmegen Centre for Molecular Life Sciences, Department of Nephrology, Radboud University Nijmegen Medical Centre, Nijmegen, The Netherlands; Massachusetts General Hospital, Howard Hughes Medical Institute, United States of America

## Abstract

Epigenetic information, such as parental imprints, can be transmitted with genetic information from parent to offspring through the germ line. Recent reports show that histone modifications can be transmitted through sperm as a component of this information transfer. How the information that is transferred is established in the parent and maintained in the offspring is poorly understood. We previously described a form of imprinted X inactivation in *Caenorhabditis elegans* where dimethylation on histone 3 at lysine 4 (H3K4me2), a mark of active chromatin, is excluded from the paternal X chromosome (Xp) during spermatogenesis and persists through early cell divisions in the embryo. Based on the observation that the Xp (unlike the maternal X or any autosome) is largely transcriptionally inactive in the paternal germ line, we hypothesized that transcriptional activity in the parent germ line may influence epigenetic information inherited by and maintained in the embryo. We report that chromatin modifications and histone variant patterns assembled in the germ line can be retained in mature gametes. Furthermore, despite extensive chromatin remodeling events at fertilization, the modification patterns arriving with the gametes are largely retained in the early embryo. Using transgenes, we observe that expression in the parental germline correlates with differential chromatin assembly that is replicated and maintained in the early embryo. Expression in the adult germ cells also correlates with more robust expression in the somatic lineages of the offspring. These results suggest that differential expression in the parental germ lines may provide a potential mechanism for the establishment of parent-of-origin epigenomic content. This content can be maintained and may heritably affect gene expression in the offspring.

## Author Summary

Epigenetic information such as parental imprints can be transmitted along with genetic information through the germ line from parent to offspring. Recent reports show that histone modifications marking developmentally regulated loci can be transmitted through sperm as a component of this information transfer. How the information that is transferred is established in the parent and maintained in the offspring is poorly understood. Here we show that expression in the parental germ line can influence the establishment of information that is then replicated and maintained in the early embryo, suggesting a potential mechanism for the establishment of parent-of-origin epigenomic content.

## Introduction

The information transferred by gametes from parent to offspring is not limited to that encoded in DNA; epigenetic information is also an important component of cross-generation inheritance [1]. How this information is established in the parent and stably maintained in the offspring is poorly understood. The importance of this information is revealed in developmental diseases that result from defective genomic imprinting, in which defective epigenetic information establishment in the parental germ line can cause abnormal somatic gene expression in the offspring [2]. Although this is limited to parent-to-offspring inheritance, recent studies suggest that epigenetic abnormalities in the parental germ line can cause heritable defects across many generations [3], [4]. The germ line therefore not only protects and distributes genetic information, but may also identify and regulate what epigenetic information is “proper” and heritable through subsequent generations.

A dramatic example of imprinting in mammals is imprinted X chromosome inactivation (iXi), in which the paternal X is preferentially inactivated prior to implantation in mammals [5]. iXi is sustained only in the placental tissues of eutherians, but is also observed in embryonic lineages in marsupials [5], [6]. Unlike most genomic imprints, iXi does not require the maintenance DNA methyltransferase Dnmt1 [7]–[9]. It does however require repressive histone modifications such as H3K9me and H3K27me established by the Polycomb group histone methyltransferases [10]–[12]. These features are consistent with the theory that histone modifications are the more conserved imprinting mark, as DNA methylation is not associated with imprinting phenomena in worms or flies, for example, and yet epigenetic imprinting phenomena have been observed in these organisms [13]–[15].

What “marks” the paternal X for iXi? One mechanism that is unique to the paternal X is meiotic sex chromosome inactivation (MSCI). MSCI targets the XY chromosome pair for significant transcriptional repression during male meiosis. This is thought to be due to the largely unpaired/unsynapsed status of these chromosomes, which renders the X and Y targets for a process generally termed Meiotic Silencing [16]. This correlation between MSCI and iXi has not gone unnoticed, and debated models linking these processes have been proposed [17], [18]. Regardless, it is clear that in mice and marsupials, passage through spermatogenesis imparts an imprint that selectively renders the X prone to early repression in the offspring, while passage through oogenesis prevents this.

The X chromosome is also condensed and transcriptionally inert during *C. elegans* spermatogenesis and, as in some mammals, the paternal X (Xp) is initially inactive in the early embryo [13], [19], [20]. This iXi in *C. elegans* consists of a near complete absence of most “active” histone H3 modifications on the Xp, a unique status that is stable through early cell divisions, becoming less obvious by ∼24 cells [13]. The absence of specific H3 modifications on the Xp implies that *de novo* zygotic chromatin assembly on the Xp is somehow uniquely and heritably refractory to addition of these marks during early stages. This information is clearly epigenetic in nature: it is only imparted to an X that has passed through spermatogenesis and not to a genetically identical chromosome encountering oogenesis. Interestingly, the Xp is “imprinted” whether it passed through spermatogenesis in an XX hermaphrodite or an XO male. That is, the pairing status of the X going through spermatogenesis does not affect imprint establishment. This seems to omit a contiguous linkage between this process and meiotic silencing mechanisms. The only determinant seems to be whether the X chromosome went through spermatogenesis before arriving in the zygote, independent of whether that spermatogenesis occurred in an XX hermaphrodite or an XO male.

A key difference between the Xp and the rest of the chromosomes in the embryo, including the oocyte-derived Xm, is a significant difference in their respective transcriptional activities in the parental germ lines. The X chromosome appears largely transcriptionally inactive in pre-meiotic and early to middle stages of meiosis in both sexes. This is likely due to the paucity of X-linked genes expressed in germ cell stages common to both sexes, which itself may be an evolved consequence of meiotic silencing mechanisms [21], [22]. The X further lacks spermatogenesis-specific genes, and thus remains largely inactive throughout sperm development in both males and larval hermaphrodites [20], [22]. In contrast, oogenesis-enriched loci are well represented on the X and this chromosome becomes active during oogenesis [20], [22]. Thus the X's from egg and sperm arrive into the zygote with significantly different transcriptional histories.

A histone modification that is associated with transcription is histone H3 lysine 4 methylation (H3K4me). H3K4me deposition can result from active transcription, and it has been implicated in providing a heritable and trans-generational memory of where transcription has occurred in the genome [23]. H3K4me has been implicated as playing an important role in differential DNA methylation, as this mark can interfere with *de novo* methylation *in vitro*, and mutations in the H3K4 demethylase KDM1b lead to defective maternal imprint establishment *in vivo* [24], [25]. It is thus possible that transcription-coupled addition of H3K4me, or its addition by other mechanisms in the parental germ line can influence the establishment of epigenetic imprints inherited by the offspring. Likewise, the absence of this mark could also have consequences for epigenetic inheritance. Importantly, H3K4me is largely absent from Xp chromatin in *C. elegans* spermatogenic germ cells, and its continued absence in the embryo is what defines iXi in this organism [13].

We therefore hypothesized that the epigenetic imprint of the Xp in the offspring is a passive consequence of its transcriptional quiescence, which implies the equally interesting idea that transcriptional activity in the adult germ line may influence the epigenetic information that is inherited by the offspring. This also implies that differential assembly of chromatin, such as that imposed by transcription in the parental germ cells, may survive gametogenesis, be transferred intact into the offspring, and be maintained in early embryogenesis. In order to test this, we further characterized chromatin assembly in the adult germ line and the heritability of epigenetic information through sperm, and studied the connections between gene activity in the parental germ line and the patterns of chromatin modifications that are maintained in the zygote.

## Results

### Sex body formation and imprint establishment are X DNA autonomous

**Figure 1:**
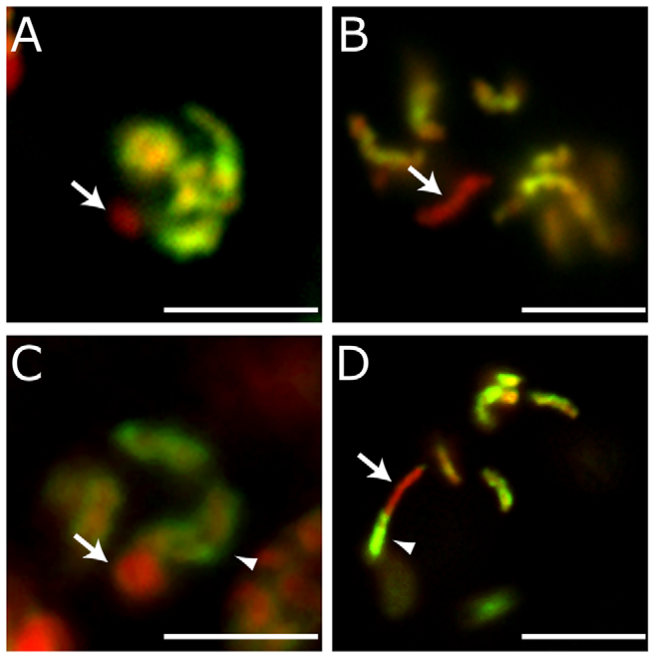
Sex body formation and imprint establishment are X DNA autonomous. The male X chromosome lacks H3K4me2 and forms a dense, sex body-like, ball in wild-type pachytene nuclei (A, arrow), and continues to lack H3K4me2 after chromatin assembly in the zygote (B, arrow) (one cell embryo shown). In L4 hermaphrodite spermatogenesis *mnt12* pachytene nuclei (C), one half of the X:IV fusion chromosome lacks H3K4me2 and forms a dense ball (arrow) while the other half remains elongated and accumulates H3K4me2 (arrowhead). In *mnt12* zygotes (D), only half of the fusion chromosome lacks H3K4me2 (arrow) while the other half accumulates H3K4me2 (arrowhead) (one cell embryo shown). H3K4me2 (green), DAPI (red). Scale bars, 5 um.

The X chromosome in *C. elegans* is largely devoid of genes that are expressed in spermatogenic germ cells as well as genes that are enriched for expression in both oogenic and spermatogenic germ cells [22]. Additionally, a number of examples have been reported in which essential loci with X- and autosomal-linked paralogs exhibit germ cell-specific defects when only the autosomal copy is defective, suggesting that only the autosomal copy is active in germ cells [26], [27]. Thus the consensus in published data suggests there is little need for transcription from the X chromosome during any stage of spermatogenesis. Indeed, few histone marks found in active chromatin are detected on X chromatin during spermatogenesis, and the X chromosome(s) become highly condensed relative to autosomes during both XO and XX spermatogenesis (Figure 1A, male pachytene nuclei with X chromosome (arrow), and [19], [20]). This premature condensation, along with the absence of H3 histone modifications correlating with transcription, is reminiscent of XY-sex body formation in mammalian spermatogenesis [28]. If X inactivity during spermatogenesis is a passive consequence of X chromosome sequence content, then attachment of X sequence to an active autosome should not affect either condensation or accumulation of active histone marks, such as dimethylated H3K4 (H3K4me2). This is indeed the case, as animals carrying a fusion of chromosomes IV and X (*mnT12*) exhibit a chromosome with both autosome and X chromosome structural characteristics that are limited to each respective half. In wild-type males (or wild-type hermaphrodite L4 larvae) undergoing spermatogenesis, the X chromosome in pachytene lacks H3K4me2 and forms a condensed ball reminiscent of the XY body in mammalian spermatogenesis (Figure 1A arrow and [19], [20]). Strikingly, in *mnt12* hermaphrodite L4 larvae undergoing spermatogenesis, one half of the fusion chromosome lacks H3K4me2 and is highly condensed (Figure 1C arrow), while the other half remains elongated and is decorated by H3K4me2 (Figure 1C arrowhead). These data suggest that the transcriptionally quiescent sex body-like structure formed during spermatogenesis is autonomous to the DNA content of the X-chromosome.

We next looked at this fusion chromosome in the early embryo. The *mnt12* chromosome is easily identifiable cytologically because it is approximately twice the length of the rest of the chromosomes [29]. When paternally inherited, this chromosome is “half-imprinted”; i.e., exhibits the characteristic absence of H3K4me2 along half its length (one cell embryo; Figure 1D). Furthermore, combined FISH-antibody analyses indicate that there is little or no spreading of H3K4me2 into the X chromosome sequence (Figure S1A). In contrast, the *mnT12* chromosome encountering oogenesis becomes fully decorated with H3K4me2, consistent with the normal representation of X-linked oocyte-expressed genes [20], and heritably maintains this status in the zygote (Figure S1B). These data suggest that the imprint in the zygote is specific for and limited to X DNA, and correlates with sex-specific gene activity in the parental germ lines.

### Mature sperm chromatin retains epigenetic information that correlates with spermatogenic transcription

**Figure 2:**
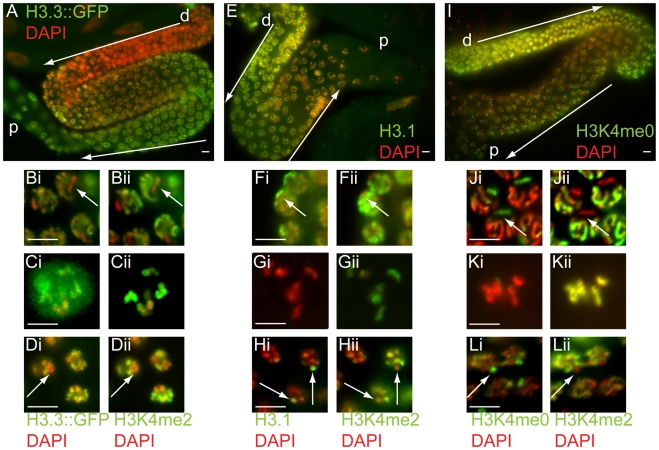
H3.3, H3.1, and H3K4me0 dynamics in oogenesis and spermatogenesis. (A–H) Comparison of H3.3::GFP (A, Bi–Di) to H3K4me2 (Bii–Dii), and H3.1 (E, Fi–Hi) to H3K4me2 (Fii–Hii), in hermaphrodite germ cells. (A) H3.3 is low in the distal nuclei of the gonad (d), and accumulates as nuclei progress towards the proximal (p) end. H3.3::GFP is absent from X in pachytene nuclei (Bi; arrow), identified by lack of H3K4me2 (Bii). H3.3::GFP is present on all chromosomes in mature oocytes (Ci), as is H3K4me2 (Cii). H3.3::GFP is absent from X's in larval hermaphrodite (spermatogenic) pachytene nuclei (Di; arrow), coincident with the absence of H3K4me2 (Dii). (E) H3.1 is high in the distal region of the gonad (d), and is depleted as nuclei progress towards the proximal (p) end. H3.1 is enriched on the X in pachytene nuclei (Fi; arrow) as identified by lack of H3K4me2 (Fii). H3.1 is low on all chromosomes in mature oocytes (Gi), while H3K4me2 is abundant (Gii). H3.1 is enriched on the paired X's in larval spermatogenic pachytene nuclei (Hi; arrows) as identified by the absence of H3K4me2 (Hii). (I, Ji–Li) X chromosome enrichment for H3K4me0 in male germ cells. (I) In both male and hermaphrodite (not shown) germ cells, H3K4me0 is high in the distal region of the gonad (d) and is depleted as nuclei progress towards the proximal (p) end. H3K4me0 is enriched on the X in pachytene hermaphrodite nuclei (Ji; arrow), as identified by lack of H3K4me2 (Jii). H3K4me0 is low on all chromosomes in mature oocytes (Ki) while H3K4me2 is abundant (Kii). H3K4me0 is enriched on the X's in larval hermaphrodite (spermatogenic) pachytene nuclei (Li; arrow) as identified by lack of H3K4me2 (Lii). Antibodies as indicated (green) with DAPI counterstain (red). Scale bars, 5 um.

**Figure 3:**
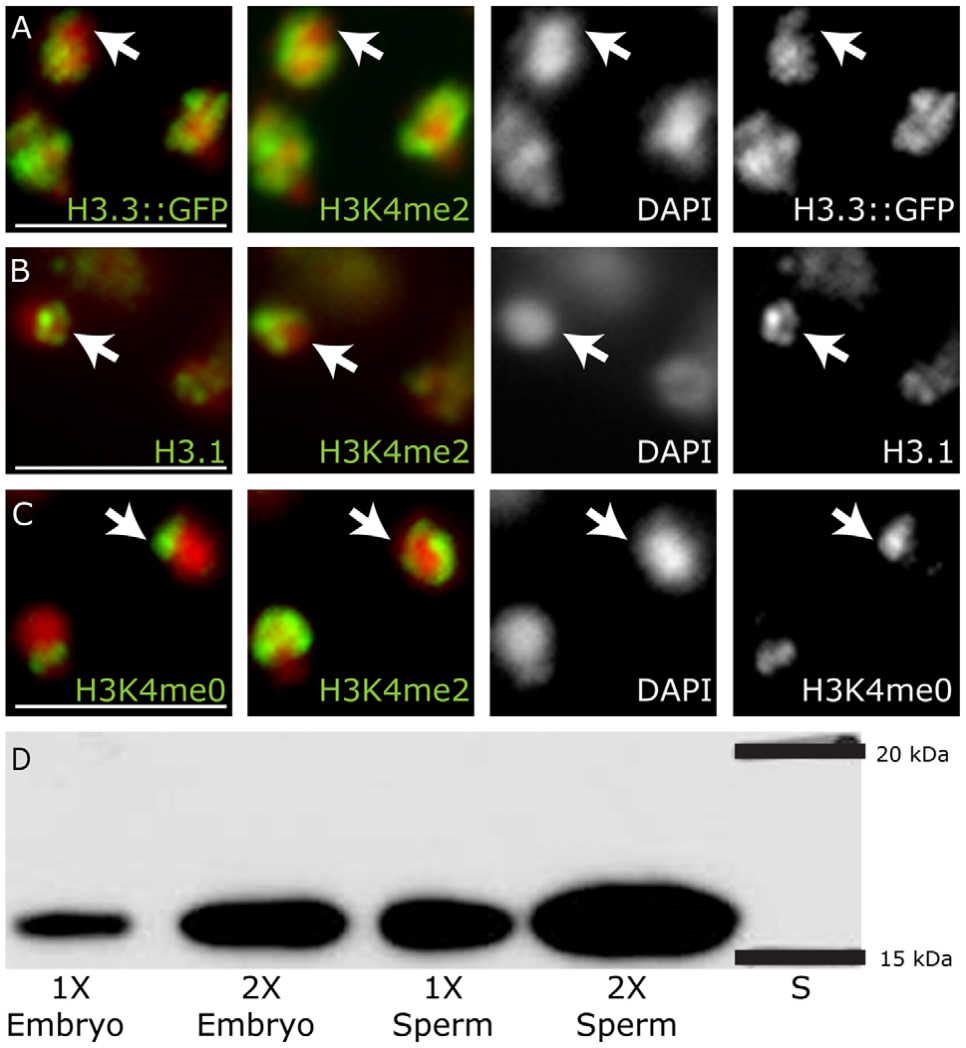
Mature sperm chromatin retains epigenetic information established in meiosis. Antibody staining for H3.3::GFP, H3.1 and H3K4me0 compared to antibody staining for H3K4me2 in mature hermaphrodite spermatids. (A) H3.3::GFP has substantial overlap with H3K4me2, but is depleted from the X region (arrow), which lacks H3K4me2. In contrast, both H3.1(B) and H3K4me0 (C) are enriched on the X chromosome, marked by a lack of H3K4me2 (arrows). Antibodies (green) with DAPI (red). Scale bars, 5 um. (D) H3 is abundant in mature sperm chromatin at levels near that of embryonic chromatin by western blot analysis. Lanes were loaded with DNA equivalents as indicated. 1) embryo 1X DNA, 2) embryo 2X DNA, 3) sperm 1X DNA, 4) sperm 2X DNA.

Histone variant H3.3 incorporation correlates with transcriptionally active chromatin and other modes of chromatin remodeling, and is reported to be enriched on the XY body and unpaired chromatin targeted by MSCI in mammals [30], [31]. In contrast, and as previously reported, a *C. elegans* H3.3::GFP (HIS-72::GFP (*zuIs178*)) expressed in germ cells is largely absent from the X but accumulates on the autosomes in pachytene nuclei during both oogenesis and spermatogenesis in *C. elegans* [32]. Additionally, HIS-72::GFP accumulates on the X in mature oocytes ([32] and Figure 2B–2D). HIS-72::GFP is known to be present in haploid spermatids and thus survives sperm chromatin condensation [32]. We further observed, however, that whereas the rest of the chromosomes contain H3.3 in mature sperm, the X remains devoid of this variant (Figure 3A). The genomic distribution of H3.3 within sperm chromatin thus grossly overlaps with the general pattern of its distribution during gametogenesis.

Although HIS-72::GFP is largely and uniquely absent from X chromosomes in spermatocytes, this does not rule out an enrichment for other H3 variants that exist in the C. elegans genome [32]. Incorporation of H3 variants outside of DNA replication involves the replacement of the canonical S-phase specific histone H3.1. We therefore tested whether H3.1 was being replaced on the X at any stage in germ cells of either sex. Using a monoclonal antibody specific for H3.1 [33] we observed that H3.1-specific staining was enriched on all chromosomes in the proliferating region of the gonad, as expected (Figure 2E). In the pachytene region of the hermaphrodite gonad H3.1 was progressively lost from the autosomes but persisted on the X chromosome (Figure 2F). However, as the germ cells progressed into oogenesis, H3.1 on the X chromosomes became reduced to levels indistinguishable from autosomes (Figure 2G). This is consistent with transcriptional quiescence of the X during meiosis and its activation during oogenesis, as previously reported [13], . In spermatogenesis, we saw similar staining in the mitotic (not shown) and pachytene regions of the gonad (Figure 2H). In contrast to oogenesis, H3.1 persisted beyond pachytene, and could be detected in a specific region in the nuclei of mature sperm (Figure 3B). We presume this to be the X, since in addition to enrichment in H3.1 the region also lacked H3K4me2 (below). These data are reciprocal of the H3.3 data, and are consistent with X chromatin being transcriptionally inactive and comparatively free of large-scale H3 remodeling throughout spermatogenesis. The male X thus uniquely retains substantial levels of H3.1 that were incorporated during pre-meiotic replication. In contrast, during oogenesis the X becomes transcriptionally active, and H3.1 is largely replaced, presumably by H3.3. Therefore the general deposition pattern of histone H3.3, as a mark of chromatin activity, is retained during spermatogenesis and is carried into the offspring with the paternal DNA.

It is possible that the enrichment of other H3.3-like variants may not have been detected in our assay. We tested deletion mutations of *C. elegans* H3.3-related variants *his-69* and *his-70* and observed no defects in X chromatin dynamics in either the adult or embryo (not shown). These H3.3 variants are thus unlikely to be specifically accumulating on the X chromosome or contributing to imprint establishment during spermatogenesis.

These data show that an X going through spermatogenesis is largely refractory to transcription-dependent or other H3 remodeling activities, presumably as a passive consequence of the paucity of genes on this chromosome that are active in spermatogenic germ cells. As a result, histone H3.1 incorporated during pre-meiotic S-phase persists in Xp chromatin in sperm. Conversely, patterns of histone variant incorporation that result from transcription or other processes appear to persist in other regions of the genome in sperm.

We next asked whether histone modifications such as H3K4me2 are present in mature *C. elegans* sperm, as has been recently reported in mammals [34], [35]. We detected substantial levels of both H3K4me2 and unmodified H3K4 (H3K4me0) in purified haploid spermatids by both Western blot analyses and immunofluorescence (Figure 3 and data not shown). Furthermore, we found that the H3K4me2 that is retained in sperm nuclei is strikingly excluded from one region in spermatid nuclei (Figure 3, arrow). Co-staining with an antibody that specifically recognizes unmodified H3K4 (H3K4me0) gave the reciprocal pattern (Figure 3C). As this pattern is consistent with what is observed for the X chromatin in all earlier stages of spermatogenesis (Figure 2L), we conclude that this region is the X chromosome. This indicates that histone H3 not only remains associated with DNA during sperm chromatin compaction, but also retains chromosomal epigenetic patterns placed during spermatogenesis. Thus, the X chromosome arriving from spermatogenesis is enriched, relative to both autosomes and an X going through oogenesis, in histone H3.1 molecules that contain few marks of transcriptional activity. Conversely, the autosomes arriving via sperm carry into the offspring both H3.3 and histone marks accumulated during parental gene activity. These experiments also show that the failure of the Xp to be recognized by antibodies against modified H3K4 is not due to epitope masking or enrichment for N-terminally cleaved forms of H3 that have recently been reported [36].

**Figure 4:**
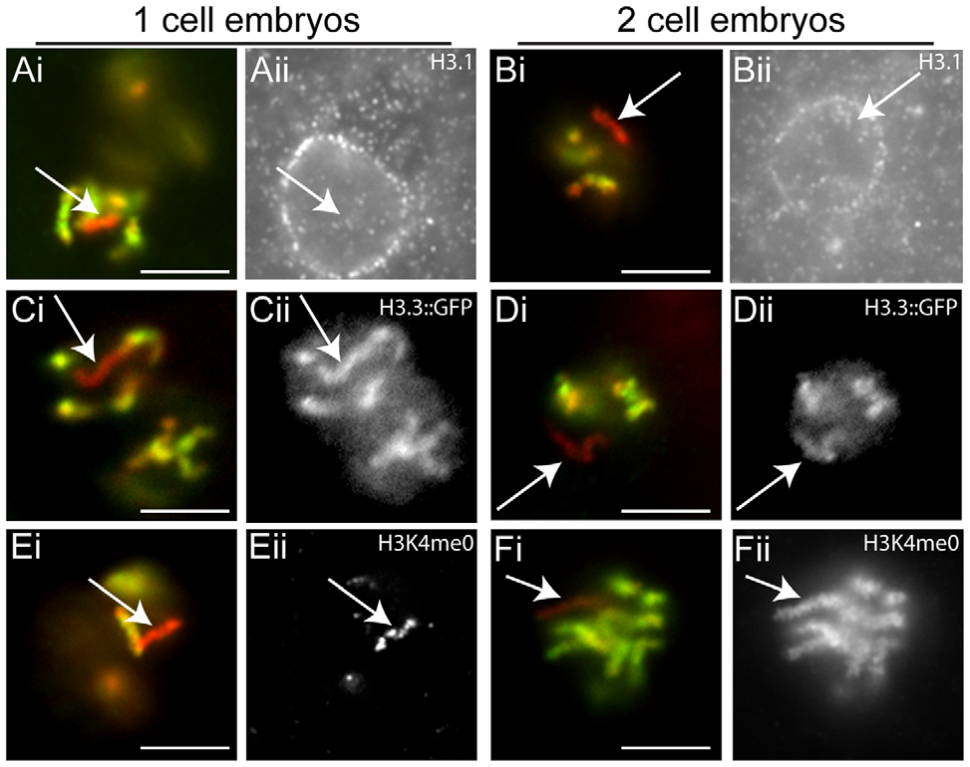
H3.1, H3.3, and H3K4me0 dynamics in the early embryo. In all image sets, a merged image of H3K4me2 (green) and DAPI (red) are shown, while the adjacent gray scale image shows a separate channel corresponding to co-staining with antibodies against H3.1, H3.3, or H3K4me0 as indicated. (Ai) In a one-cell embryo, all chromosomes except the Xp (arrow) incorporate H3K4me2 (green), but very little H3.1 is present on any chromosome (Aii). In 2 cell embryos low levels of H3.1 are detected on all chromosomes, including the Xp (Bi; arrow). Maternally provided H3.3:GFP is immediately incorporated into all sperm chromosomes, including the Xp, in the one cell stage (Cii; arrow), and thereafter (Dii nuclei from two cell embryo). Despite the incorporation of maternal H3.3, the autosomes retain H3K4me2 and the Xp (Ei; arrow) still lacks this modification, and is enriched for H3K4me0 relative to the autosomes (arrow; Eii). By the 2 cell stage and thereafter (Fii and not shown) all chromosomes (including the Xp; arrow), have incorporated high levels of H3K4me0 (Fii), but the Xp still excludes H3K4me2 (Fi; arrow). Scale bars, 5 um.

Histone H3 and its marks remain highly abundant in purified sperm chromatin, and thus the paternal chromatin enters the egg carrying substantial epigenetic information (Figure 3D). However, extensive post-fertilization remodeling of the paternal pronucleus by H3.3 occurs in many organisms, and such remodeling could significantly interrupt the heritability of sperm chromatin [33], [37]. Indeed, H3.3 (e.g., HIS-72::GFP) that is retained in mature sperm of *C. elegans* appears to disperse after fertilization, presumably through substantial incorporation of maternal H3.3 into the sperm pronucleus [32]. This indicates that as in other organisms, there is dynamic histone H3 mobilization into and out of the paternal chromatin during sperm pronuclear decondensation in the *C. elegans* zygote. These dynamics appear to precede DNA replication in the zygote. Importantly, little H3.1 is detected on any chromosome by immunofluorescence in early (1–8 cell) embryos (Figure 4Aii and 4Bii). As expected, H3.1 gradually accumulates during replication, but is not strongly apparent by immunofluorescence on any chromosome until after the 8 cell stage, after which it is more robustly detected on all chromosomes (data not shown). This suggests that the bulk of the H3 dynamics in the zygote initially involve deposition of H3.3, as observed in other species, and is largely replication independent and maternal in origin [32]. Importantly, the Xp is not noticeably resistant to H3.3 incorporation, and thus not refractive to post-fertilization histone dynamics (Figure 4Cii and 4Dii). Despite the similar H3 dynamics, the Xp maintains its relative enrichment for H3K4me0 through sperm decondensation and the first S-phase, when the autosomes also become increasingly enriched in H3K4me0 due to substantial incorporation of unmodified H3/H3.3 in all chromosomes (Figure 4Eii). Furthermore, after subsequent rounds of DNA replication and histone incorporation (by the 2-cell stage and beyond), anti-H3K4me0 signals on the Xp are indistinguishable from the other chromosomes (Figure 4Fii). However, in spite of substantial *de novo* incorporation of H3, the Xp remains uniquely devoid of H3K4me2. Importantly the autosomes and Xm maintain their H3K4me2 enrichment, established in the parental germ line, despite the significant post-fertilization H3 dynamics that we observe for these chromosomes. The H3K4me2 we observe at these early stages is likely due to maintenance of parental chromatin patterns, rather than *de novo* transcription-dependent establishment, as there is little or no zygotic transcription at these early stages [38]–[40].

These observations indicate that the chromatin assembled *de novo* in the zygote does not grossly perturb a significant amount of the epigenetic information that is carried by pre-existing histones in gamete chromatin. This implies that the unique chromatin status of the Xp could be due to an absence of an instructive template that is present on the Xm and autosomes, i.e. the epigenetic information retained during assembly of chromatin in the zygote is guided by pre-existing information present in the gamete from which it arrived. The establishment of this template could conceivably be influenced by transcriptional activity in the parent, and retained in the zygote by transcription-independent processes.

### Transcriptional activity in adult correlates with chromatin status in the embryo

**Figure 5:**
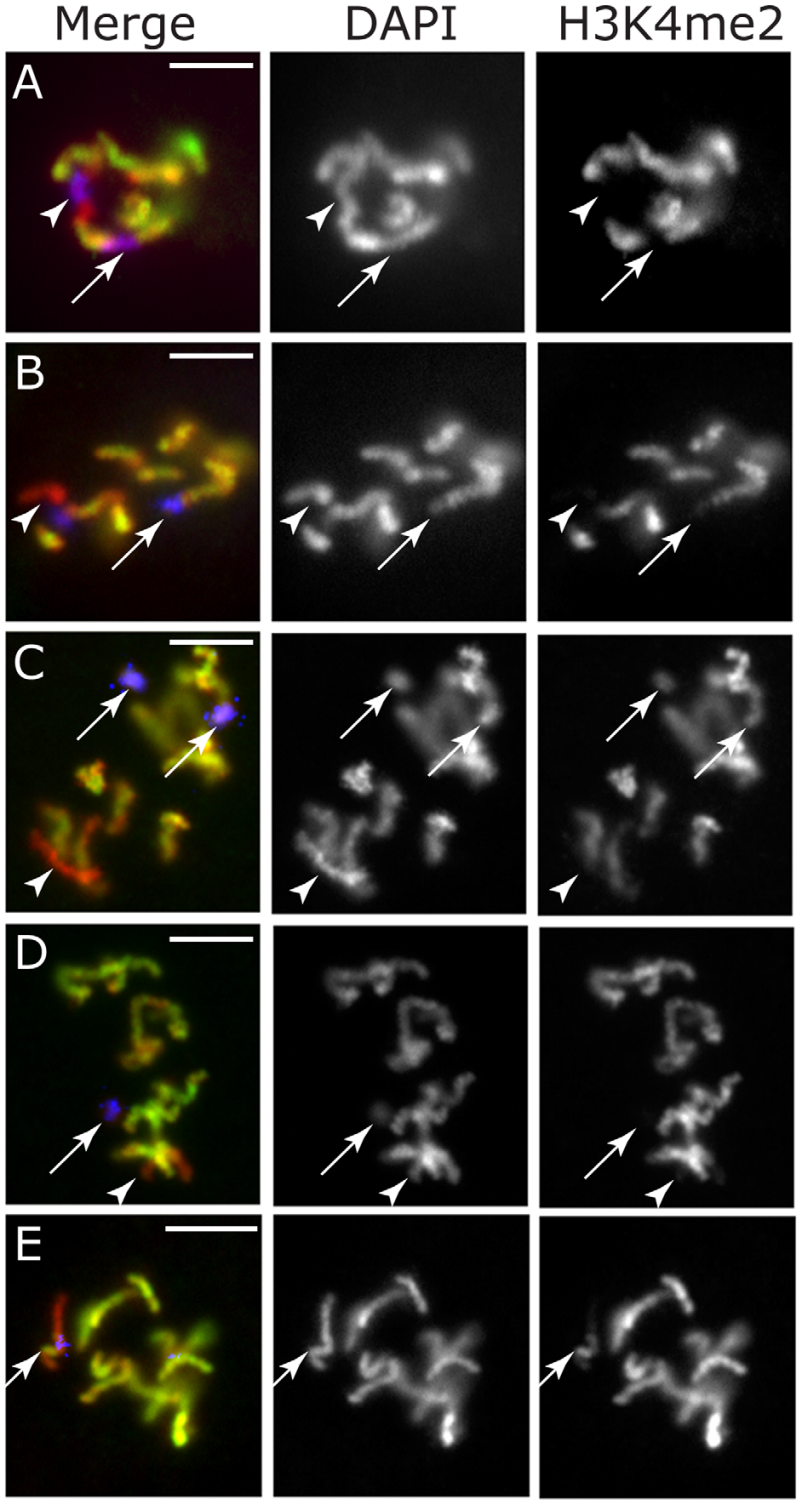
Transcriptional activity in parental germ cells influences chromatin assembly in the zygote. (A–E) Chromosomes and transgenic arrays in 1–2 cell embryos with DAPI (red), antibody against H3K4me2 (green), and DNA FISH marking the transgene (blue). Arrowheads in all panels mark the Xp; arrows mark the transgenes. (A) X-linked, germline silent *pes-10::GFP* transgene lacks H3K4me2 on Xm (arrow) in addition to Xp (arrowhead) in embryos. (B) LG V-linked, germline silent *mIs10* transgene (arrow) lacks H3K4me2 in embryos. (C) Germline expressing Ex1336 extra-chromosomal transgene (arrows) accumulates H3K4me2 in embryos. (D) Same transgene as in (C), but lacking adult germline expression and lacks H3K4me2 in embryo (arrow). (E) X-linked, germline expressing *his-24::GFP* transgene accumulates H3K4me2 on Xp in embryo (arrow). Scale bars, 5 um.

Transcriptional regulation in the germ line is poorly understood in most organisms including *C. elegans*, presenting a major challenge to experimentally modulating the transcriptional activity of endogenous loci. We therefore tested transgenes that exhibit differential germ line transcription properties and chromosome linkage to correlate epigenetic status in adult germ cells with that in the early embryo. We first examined a strain carrying an X-linked repetitive transgene with a GFP reporter driven by a soma-specific promoter [*him-5(e1490);*axIs36 (pes-10::GFP, *dpy-20(e1282)*] [41]. This reporter is not normally active in the germ line of either sex, and as reporter transgenes are typically silent in the germ line of *C. elegans*, no aberrant or ectopic GFP expression was detected in germ cells of this strain (not shown). This transgene is devoid of H3K4me2 during meiosis in the parental germ cells (Figure S2). Combined H3K4me2 antibody/DNA fluorescence *in situ* hybridization (FISH) analyses showed that H3K4me2 remained strikingly depleted from the transgene region on the Xm in embryos (Figure 5A). Importantly, the absence of H3K4me2 on the Xm transgene was observed through multiple rounds of cell division in the embryo until GFP became detectable in lineages where the *pes-10* promoter is active (8–24 cells; not shown). H3K4me2 remained depleted from the transgene in lineages where the promoter is not active until at least the 50 cell stage (not shown). The establishment of a heritable chromatin state in the embryo that correlates with transgene expression in the parental germ line is thus not specific to the sex of the germ line.

We next asked if the absence of H3K4me2 on germ line silent transgenes is limited to X-linked loci by examining a repetitive transgene construct carrying a similar GFP reporter to the transgene above, but integrated on chromosome V (*mIs10* V). In early embryos from this strain we could identify the imprinted Xp, which lacked H3K4me2 (Figure 5B arrowhead), as well as two autosomes exhibiting stripes lacking H3K4me2, which overlapped with the FISH signals detecting the transgene (Figure 5B arrows). We observed similar results for several other germline inactive transgenes (Figure S3, and Table 1). These and the above data together indicate that the absence of H3K4me2 in embryonic chromatin correlates with a lack of adult germline expression rather than germline sex or chromosome linkage.

**Table 1:** Germline expression and H3K4 methylation in different transgenes.

Strain	Transgene Name	Transgene Constructs	Chromosome	Germline Expression	H3K4me2 in Pachytene	H3K4me2 in Embryos
N2 (XO)	*n.a.*	n.a.	X	no	no	no
N2	*n.a.*	n.a.	A	yes	yes	yes
SP646 (mnt12)	*n.a.*	n.a.	X/A	no/yes	no/yes	no/yes
JH103	*axIs36*	*pes-10::GFP, dpy-20*	X	no	no	no
PD4793	*mIs10*	*pes-10::GFP, myo-2::GFP, gut::GFP*	V	no	no	no
KW1336 (*let-858*)	*Ex1336*	*let-858::GFP, rol-6*	Ex	yes	yes	yes (71%)
KW1336 outcross	*Ex1336*	*let-858::GFP, rol-6*	Ex	no	no	no (72%)
IN373	*dtIs372*	*his-24::GFP, rol-6*	X	yes	yes	yes
PD3861	*ccIn3861*	*pha-1 (+) unc-54p::GFP*	V	no	no	no
mes-4 (bn85)/DnT1	*qIs50*	*myo-2::GFP, pes-10::GFP, F22B7.9::GFP*	V	no	no	no
PD7271	*ccEx7271*	*let-858::GFP, pha-1 (+)*	Ex	no	no	no
DG1575	*tnIs6*	*lim-7::GFP, rol-6*	X	no	no	no
CB1489	*ccIn4810*	*lmn-1::GFP*	X	no	no	no
PD4251**	*ccIs4251*	*myo-3p::nuclear GFP,myo-3p::mitochondrial GFP, dpy-20*	I	no	35% yes **(N = 65)	47% yes **(N = 38)
KW1864*	*ckIn2*	*his-73::GFP, rol-6*	X	low sperm only*	no	no
TJ375	*gpIs1* [*hsp-16.2::GFP*]	hsp-16-2::GFP::unc-54 3′ UTR	A	late pachytene in ∼20 nuclei	no***	no***

Expression of the GFP reporter in adult germ cells is compared to the enrichment of H3K4me2 in the transgene chromatin in the offspring.

*The GFP expression in the KW1864 transgene is only detected in post-meiotic sperm (T.M. Edwards and W. Kelly, unpublished).

**The PD4251 transgene has been selected for non-mosaic expression, and does not display gamete-of-origin imprinting effects ([14], and A. Fire, pers. communication).

***Independent of heat shock treatment.

Repetitive transgenes are subjected to a number of silencing mechanisms, and could be targeted for assembly of repressive chromatin assembly in the embryo by mechanisms independent of template establishment [42]. We therefore tested whether embryonic chromatin assembled on a non-repetitive transgene correlated with its expression in adult germ cells. The KW1336 strain is homozygous for a mutation in the essential gene, *let-858*, but is rescued to viability and fertility by a non-repetitive, or “complex”, transgene carrying a *let-858::gfp* construct [42]. The germline expression of this transgene is maintained by selection, since failure to express *let-858* in germ cells results in sterile animals [42]. Importantly, in the absence of selection this transgene can become heritably silenced in germ cells, which allowed us to directly compare zygotic chromatin assembly on transgenes that are either active or repressed in parental germ cells.

In the line with parental germline expression maintained by selection, H3K4me2 was detectably present on the transgene array in 71% (n = 126) of embryos, of which many exhibited high levels of H3K4me2 comparable to autosomes (Figure 5C). We next outcrossed this line to wild-type males to remove the selective pressure to maintain germline expression, and followed the outcrossed line for several generations (Figure S4). Germline expression was maintained in the majority of outcross progeny in the first two generations; that is, the majority (93–100%) of offspring from germline-expressing adults initially also expressed the transgene in their germ line. By the F3 generation, however, only 69% of the offspring of germline-expressing F2 animals showed germline GFP, and germ cell expression was not detected in any of the offspring (F4) of the germline-expressing F3 animals (Figure S4). In all cases the silencing of the GFP reporter in germ cells, once established, was heritable in all subsequent generations. Note that germline silencing of the transgene was not linked to any genotype, as it eventually occurred in all descendants of all randomly selected outcross progeny.

We examined, in parallel, whether H3K4me2 was detected at significant levels on the transgene in the embryos of each generation (Figure S4). In all cases in which the parents exhibited germline GFP expression, 64–78% of the embryos showed H3K4me2 on the transgene. Note that the adult germline is syncytial, thus it is possible that some transgenes inherited by offspring from adults exhibiting germline GFP were not transcriptionally active in adult germ cells. The frequency of H3K4me2 on the transgene in embryos from germline-silenced adults dropped to ∼30%, independent of which generation the silencing occurred. A similar correlation was also observed for H3K4me3 (Figure S5). Oddly, this ∼30% frequency was stably maintained in silenced animals for many (>20) generations. This may suggest that a background level of H3K4me2 is stably, or stochastically, maintained on this transgene array long after removal of selection for germline expression. Importantly, the KW1336 array is composed of relatively few copies of the *let-858::gfp* reporter construct embedded in random fragments of *C. elegans* genomic DNA [42], [43]. Since we do not know the composition of the array other than the reporter sequences, we cannot know if this represents sporadic transcription in the adult germ line from the embedded genomic fragments, or DNA elements that may attract this modification through some other process. Importantly, none of the embryos from a different line carrying the same *let-858::gfp* reporter transgene, but in a germline-silenced array that lacks embedded genomic sequences, showed any detectable H3K4me on the transgene array (strain PD7271; Figure S3A [42]).

We further tested a total of 13 transgenes linked to various chromosomes and with various expression in adult germ cells, several of which correspond to genes that are normally expressed in all tissues (e.g., *let-858, his-24, lmn-1*), but in some cases exhibit germline silencing (Table 1). The persistence of H3K4me2 in the transgene chromatin in embryos generally follows the same pattern for all: transgene expression status in the parental germ cells is predictive for heritable and persistent H3K4me2 status on the transgene in the embryo. We observed three transgenes that did not fit this pattern. Two transgenes, the KW1864 and TJ375 strains, respectively, exhibited no detectable H3K4me2 in parental germ cells despite evidence of transcription, although both show unusual germline expression patterns. The KW1864 transgene is expressed post-meiotically during spermatogenesis, as detected by both RNA *in situ* and antibody staining (T.M.Edwards and W. Kelly, manuscript in preparation). TJ375 carries a heat-shock promoter that drives temperature dependent GFP expression in all somatic lineages, but in the adult germline expression is only detected in a narrow band of late pachytene cells, and the RNA is short-lived and not detectably translated [44]. We confirmed that transcription occurs in this limited pattern, but did not detect significant accumulation of H3K4me in transgene chromatin with or without heat shock (Table 1 and data not shown). The specific nature of the heat shock promoter, the late timing of expression, and/or the very limited period of activity may impinge upon the window of opportunity for establishing heritable active chromatin modifications in the germline. The correlation between these unusual expression characteristics and their inconsistent correlation with germline expression and H3K4me2 is not understood. The transgene in the third strain, PD4251, was selected for minimal mosaicism in its expression in muscle cells, and this selection for more efficient mitotic transmission and somatic expression may be connected to its higher “background” of H3K4me2 in the zygote ([14] A. Fire, personal communication). Nevertheless, these data show that a DNA segment can be differentially targeted for chromatin assembly in the embryo in a manner that appears to largely depend on its activity in the parental germ line, although other modes of H3K4me insertion in the parental germ cells may also participate. It is again important to note that chromatin assembly in the early stages examined has no apparent connection to zygotic transcription, as chromatin assembly and the H3K4me pattern maintenance occurs prior to any robust zygotic genome activation [38]–[40].

We next tested whether transcription activity on the Xp in adult germ cells was able to establish a region of heritable H3K4me2 maintenance on this chromosome in the offspring. The IN373 *(dtIs372)* strain carries a multi-copy *his-24::gfp* transgene that is integrated on the X chromosome. *his-24* encodes a ubiquitously expressed *C. elegans* H1 linker histone [45], [46]. The *dtIs372* transgene initially showed robust HIS-24::GFP expression throughout the soma, and weak expression in the germ line during both oogenesis and spermatogenesis (Figure S6A and S6B). In embryos from these animals we initially observed a distinct stripe of H3K4me2 on the Xp, which was largely limited to the transgene FISH signal (Figure 5E). During the course of these studies, this transgene became heritably silenced in adult germ cells, and thereafter the stripe of H3K4me2 coinciding with the transgene in embryonic Xp chromatin was no longer detected in this strain, confirming the correlation in both directions (Figure S7).

We also tested whether inheritance of H3K4me2 that was established in the parental germ line can affect expression of the transgene when it becomes active in the offspring. We compared somatic expression of the LET-858::GFP reporter in offspring from the parental line, in which germline expression is under selection, to that of outcrossed animals, in which the selection was removed and germline expression is lost (e.g., Figure S4). In both the parental line and outcrossed animals, inheritance frequency of the transgene is ∼70% as determined by DNA FISH**,** and the LET-858::GFP reporter is expressed in most if not all of the somatic tissues in adult animals [43]. Most (over 90%) of the embryos from germline expressing parents (either parental or outcrossed strain) had easily detected GFP-positive nuclei in most if not all somatic cells, whereas few (14%; n = 92) of embryos from germline silent parents had detectable GFP expression (Figure S8A). This is far below the frequency of array inheritance in these offspring. GFP expression was significantly weaker among embryos from the germline silent outcrossed line, and strong mosaicism of expression was also often observed (Figure S8B). This basal level of expression was consistent in offspring from the germline silenced animals for >20 generations (not shown).

**Figure 6:**
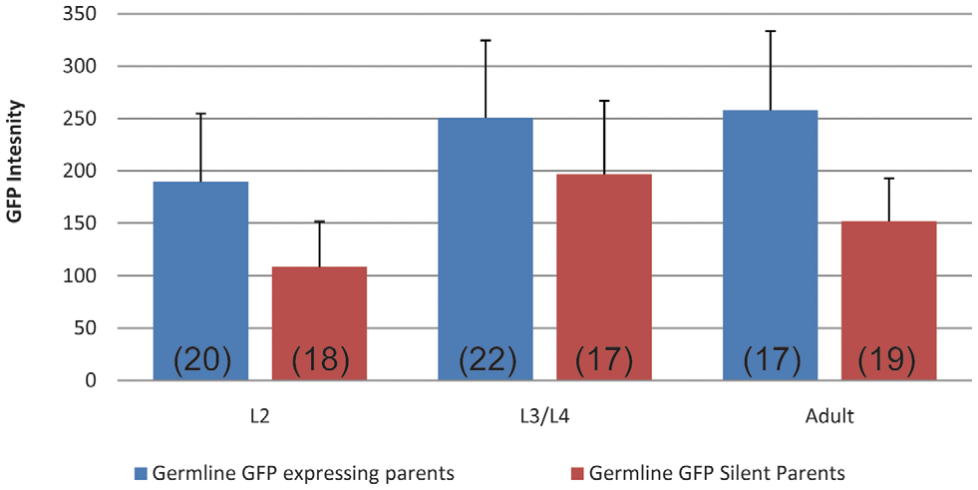
Somatic GFP intensity is higher in offspring from germline expressing than in offspring from germline silent parents. Average GFP intensity (arbitrary units) of somatic nuclei in offspring from germline GFP expressing (KW1336) parents vs germline GFP silenced (N2 outcrossed 20+ generations) parents at larval stages (L2 and L3/L4) and adults. Number of animals scored is indicated in parentheses. Error bars show standard deviation. L2 (p<0.0001). L3/L4 (p<0.009). Adult (p<0.0001).

The increased expression frequency in embryos from the germline-expressing parents, which exceeded the inheritance rate of the transgene, indicated that at least part of the GFP detected in these embryos was provided maternally. We therefore compared GFP fluorescence of the two sets of transgenic offspring at larval and adult stages. L2 larvae through adult stage offspring from germline-expressing parents still showed significantly higher levels of GFP expression compared to the germline-silenced offspring both immediately after germline silencing occurs, and after many generations of the transgene being shut down in the germline (data not shown and Figure 6). These results indicate that, at least for this transgene, expression in the adult germ line strongly correlates with enhanced somatic expression in the offspring, even in late stages of development.

It has been reported that repetitive transgenes, including several that we examined in this study, accumulate H3K9me3 during meiosis [47]. We examined H3K9me3 to see if this mark, accumulated in the parent, is also inherited by the offspring and/or correlates with H3K4me inheritance. The presence of this mark could prevent the addition of H3K4me2 to inherited chromatin in the zygote, and thus be causal to its absence. Importantly, this correlation does not exist for the Xp, which lacks both H3K9me3 and H3K4me2 in both adult germ cells and in the zygote (Figure S9). In addition, although H3K9me3 may also be inherited from gamete chromatin, there was no obvious correlation between H3K9me3 presence and H3K4me2 absence on transgenes in either adult germ cells or in embryonic chromatin. On all transgenes examined, including those that expressed in adult germ cells, we observed an enrichment of H3K9me3 on transgene chromatin in pachytene and oocyte nuclei, and this mark, like H3K4me2/3, persisted in the embryo (Figure S9Gii-S9Iii). The presence of H3K9me3 in transgene chromatin did not correlate with either expression in the parent, or the presence or absence of H3K4me2 in the embryo.

The lack of correlation between expression in the germ line and appearance or absence of H3K9me3 on transgenes, and the absence of H3K9me3 on the imprinted Xp at any stage, indicates that H3K9me3 accumulation on transgenes is due to a mechanism distinct from, and has little detectable influence on, the heritable chromatin assembly we report here. In addition, we did not observe either enrichment for, or absence of, other repressive marks (e.g., H3K27me2 or H3K27me3) on either germline-silent or germline-expressed transgenes (Figure S10 and data not shown). Indeed, abundance of these marks was indistinguishable between transgenes and autosomes in both germ line and embryo.

These data show that the establishment of heritable epigenetic information that guides chromatin assembly in the zygote is likely gene autonomous, independent of chromosomal location, and that the information established can be guided by transcriptional activity in the adult germ line.

## Discussion

Our data shows that epigenetic information in the form of histone modifications and variants, imposed during transcription and other chromatin-modifying processes in the adult germ line, can be stably carried by gametes into the offspring. This information appears to be grossly impervious to *de novo* chromatin assembly and histone dynamics in the embryo, and is maintained in the embryo in the absence of significant levels of zygotic transcription. These results suggest that sex-specific activities, including gamete-specific transcription, in the parental germ line can create parent-of-origin specific epigenetic content that may determine or guide the epigenetic information that is maintained in embryonic chromatin during development. This information content could directly affect transcriptional regulation in later development or may provide a bias for stochastic aspects of epigenetic regulation.

*C. elegans* spermatids retain histones, histone variants, and histone modification patterns that were established in the parental germ line. During spermatogenesis in plants and a wide range of animals, histones are largely replaced by protamines, small basic proteins synthesized in late stage spermatids, to facilitate sperm compaction [48], [49]. While the extent of histone displacement can vary between organisms, histone retention in spermatid chromatin is now recognized as being substantial in many organisms, as histones have been observed in the mature sperm of mammals, amphibians, *Drosophila*, and *C. elegans* [32], [49]–[53]. Importantly, histones and their modifications are retained at developmental promoters and imprinted loci in human and mouse sperm [34], [35]. Histones, and any accompanying histone modifications, surviving protamine replacement therefore have the capacity to transmit epigenetic information across generations. Our results show that sperm chromatin can retain epigenetic information that reflects prior genetic activity in the parental germ cells, and which appears to template epigenetic patterns maintained in the early zygote. Inheritance of modified histones is thus likely to be a conserved mode of epigenetic information transferred between generations, and parental transcription may therefore contribute to the content of this information [4], [33].

Recent data provides evidence for such a system. Histone H3K36 methylation, like H3K4me, is normally considered a consequence of transcriptional activity. In budding yeast, the Set2 methyltransferase tracks along with the elongating RNA polymerase holoenzyme, adding this mark within the body of transcribed genes [54]. Unlike yeast, however, metazoans have more than one H3K36 methyltransferase: in *C. elegans* this additional activity is encoded by the *mes-4* gene, which is required for germline viability [55]. Unlike Set2, MES-4-dependent H3K36 methylation appears to be a maintenance activity operating outside of ongoing transcription– it can maintain H3K36me in the bodies of genes that are transcriptionally inert [56], [57] Amazingly, the genes marked by MES-4 in embryos are specifically those that are expressed in post-embryonic germ cells, and exclude genes solely (and actively) expressed in embryonic somatic lineages [56], [57]. MES-4 system is thus maintaining, in the embryo, an “epigenetic memory” of transcription in genes that were last transcribed in adults. Intriguingly, we recently found that components of the conserved SET1/MLL H3K4-specific methyltransferase complex are essential for normal H3K4 methylation in the early embryo and in adult germline stem cells. Furthermore, these studies showed that the SET1/MLL complex is largely required for the transcription-independent maintenance of H3K4me in early embryonic chromatin, similar to the maintenance activity of MES-4 for H3K36me [58]. Therefore the heritable patterns we observed to be stably transmitted though gamete chromatin are being maintained in the zygote by an MLL-like complex. It is thus interesting to note that the MLL complex has recently been implicated as being essential for embryonic stem cell self-renewal [59].

It is clear however, that not all of the epigenetic information transferred into the zygote is stable; indeed, there is extensive chromatin remodeling and epigenetic reprogramming observed after fertilization in most species. However, some information survives and is maintained; how this maintenance is achieved is only now beginning to be identified. Whereas DNA methylation has a clear propagation intermediate and activity for maintenance (e.g., hemi-methylated DNA and DNMT1 methyltransferase, respectively), mechanisms that can maintain a memory composed of histone modifications have been less clear. The inheritance of repressive histone marks, however, has received significant attention. The well-known mitotic inheritance and propagation of H3K27 methylation by the PRC2 complex, for example, requires the H3K27me3-recognition domain of the EED subunit[60]. It is thus likely that marks such as H3K4me2 could also have analogous propagation mechanisms. Histones and their marks that are inherited from parental chromatin could “seed” an epigenetic template that is recognized and locally propagated in the absence of transcription in the zygote. This would presumably involve H3K4me recognition proteins capable of recruiting methyltransferase activities. The epigenetic signature thus retained or re-established may be maintained with meta-stable fidelity in the germ line epigenome across generations to guide and maintain germ cell function and pluripotency. In somatic lineages at each generation, however, this information would become increasingly altered during tissue-specific zygotic transcription, thereby leading to decreased pluripotency in differentiated cells.

Our results predict that epigenetic information involving H3K4me, if ectopically presented to the zygote, could become stabilized through germline passage and have functional consequences for transcription patterns in the offspring. H3K4 methylation has been directly implicated in the heritable maintenance of transcriptional activity in *Drosophila* via the Trithorax system, and more recently in *Dictyostelium* [23], [61]. Indeed, lysine 4 of H3 has been specifically implicated in developmental epigenetic “programming”, and this residue in H3.3 has been reported to be essential for fertility in *Drosophila*, although the specific role of K4 in the fertility defects has been questioned [62]–[64]. We have shown that mutations in *spr-5*, a *C. elegans* ortholog of the H3K4me2 demethylase Lsd1/Kdm1, cause a generation-dependent build-up of H3K4me2 levels in germ cell chromatin, resulting in sperm-specific transcriptional defects and ultimately germ cell failure [4]. This is strong evidence that histone modifications can become heritably stable across generations, and that the ectopic presence of H3K4me in the germline epigenome can have lasting consequences on transcriptional regulation in subsequent generations. Removal of H3K4me2 acquired during spermatogenesis from spermatid or sperm pronuclear chromatin may thus be required to prevent its inappropriate templating in the zygote and subsequent generations. How the information established at any locus is targeted for maintenance or erasure is not known in any system and remains an important question.

It is interesting that the somatic expression of the KW1336 transgene in the offspring appears to be influenced by its activity in the parental germ cells. Expression in the adult germ cells and the consequential increased inheritance of “active chromatin” may help overcome or prevent default modes of repression and may favorably bias expression in the offspring. We have not been able to test directly whether *ectopic* activation of a transgene in the germ line can cause heritable ectopic activation in offspring. However initiation of the repressed state, e.g., the spontaneous silencing of active transgenes in adult germ cells, is always heritable and appears to be permanent: such silenced transgenes have never been observed to reactivate in our lab under normal conditions, even after many generations (these studies and W. Kelly, unpublished). Indeed, the germ line appears to be preferentially poised for repression in *C. elegans*, with numerous interrelated RNAi and chromatin based mechanisms targeting exogenous DNA for silencing (e.g.,[42]). The persistence of H3K4me2 on the outcrossed *KW1336* transgene (in the absence of detectable expression in the adult) is also interesting. This could reflect persistence of a metastable chromatin state provided by genomic sequences in the in transgene chromatin that is unrelated to the regulation of the GFP reporter. Alternatively, the reporter chromatin retains a stable memory of its prior germline activity, but this is insufficient to overcome other modes of repression that target the transgene in both soma and germ line. *De novo* establishment of a heritably active state clearly has numerous obstacles in the germ line, as is appropriate for the genetic and epigenetic guardian of the species.

The relationship of iXi in the offspring to transcriptional repression in the adult germ line in both worms and some mammals is striking, irrespective of the issue of continuity of the inactive state. As in *C. elegans*, iXi in mice is meta-stable, in that it is ultimately reversed: it is stabilized in the first lineages to differentiate, the extra-embryonic tissues, but (as in *C. elegans*) becomes unstable in embryonic cells and is ultimately replaced by another dosage compensation process, random X inactivation [5], [65]. In metatherians that have been tested, iXi occurs despite the absence of an XIST locus [66]–[68]. Some forms of iXi could thus have arisen or been adapted from a mechanism similar to the templated chromatin assembly we observe in *C. elegans.* Marsupial X-linked loci silenced during meiosis are activated in round spermatids, yet still subjected to iXi in the zygote [69]. This may suggest a temporal restriction for the establishment of a chromatin state that can be interpreted by the zygote, perhaps similar to what we observed for late- or post meiotically expressed transgenes in this study.

iXi in mice does not require the maintenance DNA methyltransferase Dnmt1, but does involve repressive histone modifications [7]–[12]. These features are consistent with the theory that histone modifications may be the more ancient imprinting mark. Indeed, epigenetic imprinting phenomena have been observed in many organisms independent of DNA methylation, such as *C. elegans* and numerous insect species [13]–[15], [70]. Furthermore, H3K4 methylation has been proposed to affect the establishment of *de novo* DNA methylation, such as imprinted DNA methylation [25]. Mammalian imprinting may thus be an adaptation imposed upon more ancient, DNA methylation-independent modes of imprinted gene regulation. We propose that sex-specific incorporation of epigenetic information via transcription-linked processes (or lack thereof) in the adult germ line may be a conserved process that could guide imprinted chromatin assembly in the offspring. A sex-specific difference in transcription and/or H3K4 methyltransferase activity across a locus during gametogenesis could thus provide an underlying mechanism that contributes to imprint establishment in the germ line, and perhaps influence imprinted gene regulation during embryonic development.

## Methods

### Strains

We used standard techniques for worm maintenance and handling. We carried out all crosses and grew all worms at 20°C. We used the following strains: wild-type N2 (Bristol), mnt12 (X;IV fusion), zuIs178 [(his-72^1 kb^::HIS-72::GFP); *unc-119(ed3),* gift from S. Henikoff], *his-69(gk394), his-70* (gift from D. Chu), *him-5(e1490);*axIs36 (pes-10::GFP, *dpy-20(e1282)*), mIs10 (myo-2::GFP, pes-10::gfp, F22B7.9::gfp, V), mes-4(bn85)/DnT1 (*unc-?(n754), let-?* qIs50(myo-2::GFP, pes-10::GFP, F22B7.9::GFP), gift from S. Strome, KW1336 *unc-4(e120) let-858(cc500)* II; unc-4(e120)let-858 Ex1336 [pBK48.1 (let-858::GFP)];pRF4), Ex1336 [pBK48 (let-858::GFP)], PD7271 ((*pha-1(e2123)* III; *ccEX7271*(pBK48.1[*let-858::GFP*] and pC1 [*pha-1*(+)])), *dtIs372 (his-24*::HIS-24::GFP, X), gift from B. Conradt, PD3861 (*pha-1(e2123ts) III; ccIn3861 (pha-1* (+) *unc-54p*::GFP)) gift from A. Fire, KW1864 (*ckIn2 his-73*::GFP, *rol-6*), PD4251 (*ccIs4251* (*myo-3p*::nuclear GFP,*myo-3p*::mitochondrial GFP, *dpy-20*) *dpy-20(e1282*)), DG1575 (*tnIs6 lim-7*::GFP, *rol-6*), *ccIn4810 lamin*::GFP (X). TJ375 (*hsp-16-2::GFP::unc-54 3′ UTR*) gift from J. Priess,

### Immunocytochemistry

Whole-mount fixation and antibody staining of worms and embryos with methanol/acetone fixation was done as previously described [71]. For detection of specific histone modifications, we used the following primary antibodies at the specified dilutions: rabbit antibody to H3K4me2 (1∶500; Millipore), mouse antibody to GFP (1∶200, Millipore), mouse monoclonal #34 against H3.1 (1∶300) [33], mouse monoclonal CMA301 specific for unmodified H3K4 (H3K4me0; 1∶500; a gift from H. Kimura, Osaka University, Japan), rabbit polyclonal antibody to H3K4me3 (1∶1000 Abcam), rabbit polyclonal to H3K9me3 (1∶100 Abcam). Secondary antibodies were used at 1∶500: Alexaflour™;594 donkey anti-rabbit IgG, Alexaflour™; 488 donkey anti mouse IgG Alexaflour™;594 donkey anti rat IgG (Molecular Probes). Fluorescein-labeled antibody to digoxigenin was used at 1∶200 (Roche).

### Immunofluorescence analyses

Images were obtained using a Leica DMRA microscope outfitted with a Q-imaging Retiga-SRV Fast 1394 Camera. We acquired and processed the images with Simple PCI software. For analyses of H3K4me2 in transgene chromatin in the embryo, embryonic nuclei were optically sectioned at intervals of 0.1–0.2 micrometers to detect transgene arrays. Array chromatin in one to four cell embryos was scored blind to the genetic background of the animals as one of the following: None (-), Low (+) or Hi (++) relative to the autosomes and imprinted Xp in the same embryo.

### FISH analysis

For combined histone antibody and DNA FISH experiments, we did sequential antibody staining, and DNA FISH with a digoxigenin-labeled probe detected by a fluorescein-labeled anti-digoxigenin antibody (Roche), as previously described [13]. We carried out FISH using the digoxigenin-labeled (Roche) probe L4054 (Fire Lab vector Kit) that recognizes the sequence for GFP in transgenic worms. X-paint probe (Figure S1) was generated by digoxigenin labeling of a mixture of X-chromosome YACs (generous gift from G. Csankovszki). We observed the prepared samples and recorded and processed images as described above.

### Protein preparation and western blot

Mixed stage embryos were harvested from gravid adults by standard procedure [72]. Purified sperm were a kind gift of S. L'Hernault. To each 150 ul sample 450 ul of grinding buffer (15 mM HEPES, 10 mM KCl, 5 mM MgCl2, 0.5 mM EGTA, 15% Glycerol, 1 mM DTT, 1X complete mini EDTA-free protease inhibitor cocktail [Roche]) were added, frozen in liquid nitrogen, homogenized, re-frozen in liquid nitrogen, and thawed on ice for 15 mins. 600 ul of 2X extraction buffer (500 mM NaCl, 0.8% NP40, 10 mM HEPES (pH 7.5), 1 mM MgCl2, 1 mM DTT, 1X protease inhibitor) were added to samples, vortexed and rocked for 30 mins. at 4°C. Samples were centrifuged at 8000xg for 5 mins. at 4°C, and supernatant was removed. Pellet was re-suspended in 222 ul digestion buffer (0.4% NP40, 10 mM HEPES, 1 mM MgCl2, 1 mM DTT, 1X protease inhibitor cocktail). 30 ul of sample was sonicated in a sonicating water bath for 15 mins. and DNA concentration was quantified with a BioRad VersaFluor Fluorometer using the BioRad Fluorescent DNA Quantitation kit. Equivalent amounts of DNA for each sample were loaded and run on a 15% SDS-PAGE gel, transferred and probed with antibody for H3 (1∶5000; Abcam ab1791) detected by Goat anti-rabbit IgG HRP conjugated secondary antibody (1∶5000; Upstate Biotechnology, Inc.) and detected by chemiluminescence with the Amersham ECL Plus Detection kit.

### GFP expression analysis

KW1336 or outcrossed animals (F20+) rolling adults carrying the Ex1336 transgene were cloned, and mixed stage embryos were harvested from gravid adults by a brief (20 second) hypochlorite treatment and either harvested or allowed to develop. Embryos were then transferred to painted-well slides and examined at 100X by phase contrast and GFP using a Leica DMRA microscope outfitted with a Q-imaging Retiga-SRV Fast 1394 Camera. 1.5- to 3- fold embryos were scored + or – for the presence of detectable nuclear LET-858:GFP fluorescence. Animals were allowed to develop at 20 degrees for 36 hrs (L2's), 60 hrs (L3/L4's) or 84 hrs (adults) and mounted on 5% agrose in microbeads and images were caputured at 40X by phase contrast and GFP as above. 5–10 gut nuclei and 5 background areas were measured per animal. The average nuclear GFP intensity minus the average background intensity for each animal was calculated to give average adjusted GFP intensity. 17–22 animals were scored for each background at each time point.

## Supporting Information

Figure S1 X chromatin is only refractory to H3K4me2 when X is inactive. (A) Adult pachytene stage nucleus carrying *mnT12* IV:X fusion. H3K4me2 on LG IV chromatin does not appreciably spread into X sequences. (B) Oocyte carrying the *mnt12* IV:X fusion. The X half of the fusion chromosome has H3K4me2 at a level indistinguishable from the attached autosomal DNA. The X is transcriptionally active during oogenesis. Scale bars 5 um.
(0.64 MB TIF)

Figure S2 H3K4me2 in transgene chromatin in germ cells correlates with germline transcription. (A–E) Pachytene nuclei from adult hermaphrodites with DAPI (red), antibody against H3K4me2 (green), and transgene DNA FISH (blue). (A) X-linked, germline silent *pes-10::GFP* transgene (arrows) lacks H3K4me2, as does the rest of the X chromosome, in pachytene nuclei. (B) LG V-linked, germline silent *mIs10* transgene (arrows) lacks H3K4me2 in pachytene nuclei. (C) Germline expressing Ex1336 extrachromosomal transgene (arrows) accumulates H3K4me2 in pachytene nuclei. (D) Germline silent Ex1336 extrachromosomal transgene (arrow) in wild-type background lacks H3K4me2 in pachytene nuclei. (E) X-linked, germline expressing *his-24::GFP* transgene accumulates H3K4me2 on X in pachytene nuclei (arrow) (FISH not shown).
(3.49 MB TIF)

Figure S3 Germline repressed transgenes maintain imprinted chromatin in the early embryo. (A) Extrachromosomal array PD7271 (arrow) lacks H3K4me2 in embryo, as does the germline silent transgene ccIn3861 (B).
(0.64 MB TIF)

Figure S4 Transgene germline GFP expression versus transgene H3K4me2 in embryos. (A) Schematic of selection of animals during this experiment. At each generation animals were cloned out and allowed to lay embryos. Half of the animals were scored live for germline GFP expression and the remaining animals were dissected and their embryos analyzed by DNA FISH and anti-H3K4me2 immunofluorescence (B) % of animals LET-858::GFP fluorescence in the germline as adults at each generation (blue bars) vs. % of offspring from each generation retaining H3K4me2 on the transgene as embryos (red bars). Offspring from germline expressing parents and offspring from germline silenced parents were examined separately in the F3 and F4 generations as indicated. Number of animals scored is indicated in parentheses.
(1.00 MB TIF)

Figure S5 H3K4me3 of transgenes in germ cells and early embryo correlates with germline transcription. (A–J) Pachytene nuclei from adult hermaphrodites or one to two cell embryos with DAPI (red), antibody against H3K4me2 (green), and DNA FISH (blue). (A) X-linked, germline silent pes-10::GFP transgene lacks H3K4me3, as does the rest of the X chromosome, in pachytene nuclei. (B) X- linked *pes-10::GFP* transgene also lacks H3K4me3 on the Xm in a two cell embryo. (C) LG V-linked, germline silent *mIs10* transgene (arrow) lacks H3K4me3 in pachytene nuclei. (D) LG V-linked, germline silent *mIs10* transgene (arrow) also lacks H3K4me3 in a one cell embryo. (E) Germline expressing Ex1336 extrachromosomal transgene (arrows) does not appear to have H3K4me3 in pachytene nuclei. (F) Germline expressing Ex1336 extrachromosomal transgene (arrows) does accumulate H3K4me3 in a one cell embryo. (G) Germline silent Ex1336 extrachromosomal transgene (arrow) in wild-type background lacks H3K4me3 in pachytene nuclei. (H) Germline silent Ex1336 extrachromosomal transgene (arrow) in wild-type background lacks H3K4me3 in a one cell embryo. (I) X-linked, germline silent *his-24::GFP* transgene lacks H3K4me3 on X in pachytene nuclei (arrow). (J) X-linked germline silent *his-24::GFP* transgene lacks H3K4me3 on Xp in one cell embryo.
(2.77 MB TIF)

Figure S6 X-linked HIS-24::GFP transgene is expressed in the germline of both sexes. X-linked HIS-24::GFP is expressed in both hermaphrodite (A) and male (B) germ cells, as shown by antibody staining for GFP. Scale bars, 5 um.
(0.77 MB TIF)

Figure S7 H3K4me2 is absent from Xp in pachytene and embryos in germline silent his-24::GFP. DAPI (red), antibody against H3K4me2 (green), and DNA FISH (blue). (A) X-linked his-24::GFP transgene (arrow) in a pachytene nuclei lacks H3K4me2 when transgene is germline silent. (B) X-linked his-24::GFP transgene (arrow) in a two cell embryo lacks H3K4me2 when transgene is germline silent.
(0.81 MB TIF)

Figure S8 Transgene from germline silenced parents show reduced embryonic expression. GFP fluorescence (Ai–Ci) or DIC (Aii–Cii) microscopy of 1.5-fold to 3–fold stage live embryos. (Ai) 93% (n = 27) of embryos from KW1336 offspring from parents which express *let-858:gfp* in the germline show robust GFP expression in all nuclei. (Bi) 87% (n = 47) of offspring from outcrossed animals where germline expression of *let-858:gfp* was lost lacked any GFP positive nuclei (approximately 40% of offspring inherit the array). (Ci) Rare (13% n = 47) embryos from outcrossed parents where germline expression of *let-858:gfp* was lost with GFP positive nuclei (far right) have weaker and more variegated GFP expression than offspring from germline expressing parents (Ai). (D) % of animals expressing let-858::GFP in the germline as adults at each generation (blue bars) vs. % of offspring expressing somatic GFP as embryos (red bars). Number of animals scored is indicated in parentheses.
(1.41 MB TIF)

Figure S9 Transgenes are enriched for H3K9me3 in pachytene, oocytes, and early embryos independent of activity in the germline. Pachytene nuclei, oocytes, and nuclei from one or two cell embryos with H3K4me2 (green) and DAPI (red) (Ai–Li) or H3K9me3 (Aii–Lii) are shown. (Ai–Ci) X-linked, germline silent *pes-10::GFP* transgene (arrow) is enriched for H3K9me3 over autosomes and surrounding X-chromatin(Aii–Cii), (Di–Fi) LG V-linked, germline silent *mIs10* transgene identified by lack H3K4me2 (arrow) is enriched for H3K9me3 over autosomes and surrounding chromatin (Dii–Fii). (Gi–Ii) Germline expressing Ex1336 extrachromosomal transgene (arrows) is enriched for H3K9me3 over autosomes, particularly in oocytes and early embryos (Gii–Iii). (Ji–Li) Germline silent extrachromosomal array PD7271 (arrow) is enriched for H3K9me3 over autosomes, particularly in oocytes and early embryos (Jii–Lii).
(1.74 MB TIF)

Figure S10 H3K27me2 and H3K27me3 in transgene chromatin do not noticeably correlate with transgene expression. Oocytes with DAPI (Ai–Di) and H3K27me2 (Aii and Cii) or H3K27me3 (Bii and Dii) are shown. (Ai–Bi) Extrachromosomal germline silent transgene PD7271 (arrow) is decorated with H3K27me2 (Aii) and H3K27me3 (Bii) at levels similar to autosomes. (Ci–Di) Extrachromosomal germline expressing transgene KW1336 (arrow) is decorated with H3K27me2 (Cii) and H3K27me3 (Dii) at levels similar to autosomes. The PD7271 transgene array is repetitive and silenced; the KW1336 array is more complex and expresses in germ cells. Neither of these characteristics correlate with presence or absence of H3K27me2/3 as levels on both arrays are similar to levels observed on chromosomes in the same nuclei.
(0.81 MB TIF)
