# Supplementary figures and images for: Epigenetic Patterns Maintained in Early Caenorhabditis elegans Embryos Can Be Established by Gene Activity in the Parental Germ Cells
Source: PLoS Genet. 2011 Jun 9;7(6):e1001391. doi: 10.1371/journal.pgen.1001391 (PMC3111476; doi:10.1371/journal.pgen.1001391)

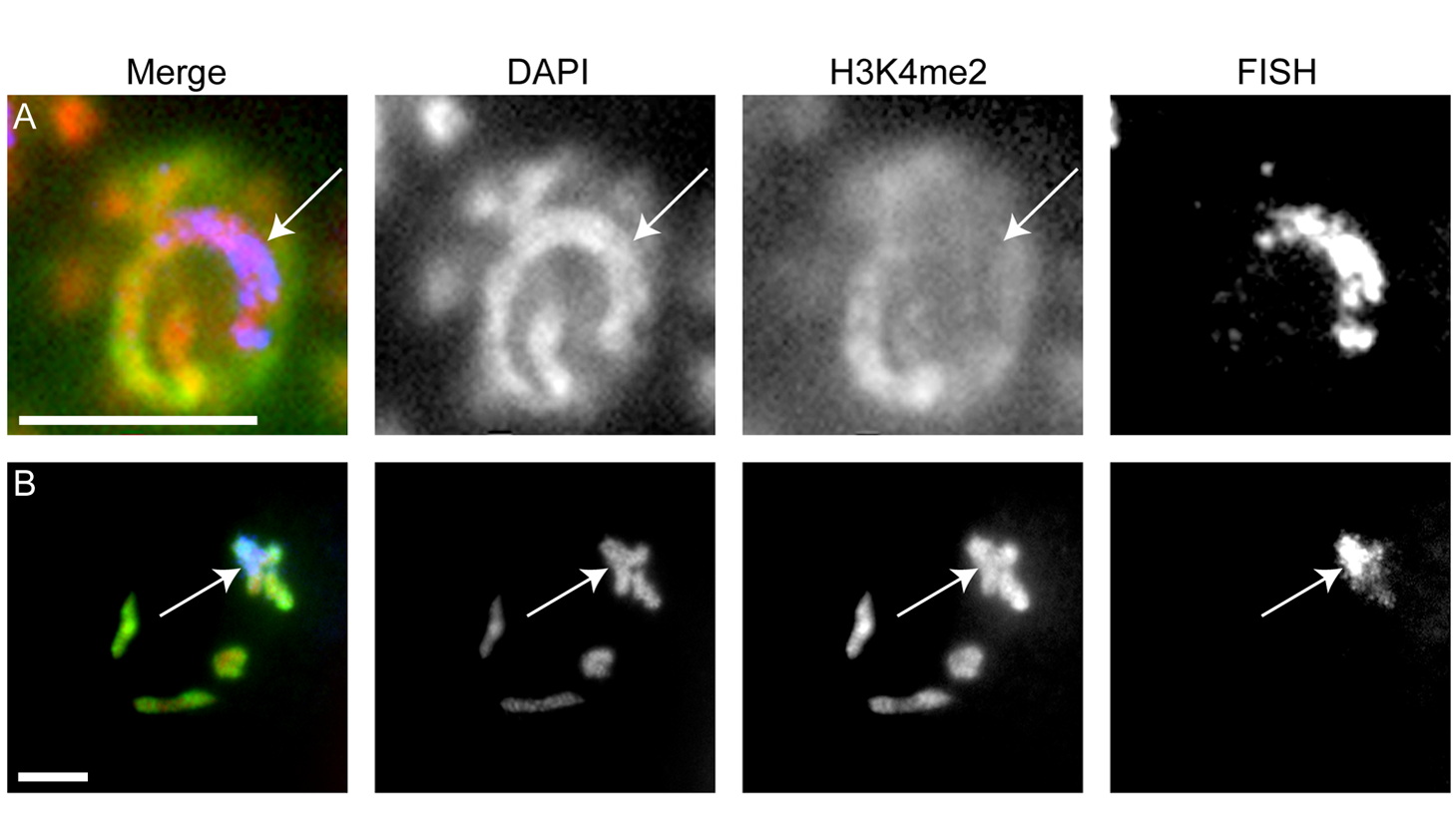

Supplement: Figure S1 — X chromatin is only refractory to H3K4me2 when X is inactive. (A) Adult pachytene stage nucleus carrying mnT12 IV:X fusion. H3K4me2 on LG IV chromatin does not appreciably spread into X sequences. (B) Oocyte carrying the mnt12 IV:X fusion. The X half of the fusion chromosome has H3K4me2 at a level indistinguishable from the attached autosomal DNA. The X is transcriptionally active during oogenesis. Scale bars 5 um. (0.64 MB TIF) [file pgen.1001391.s001.tif]

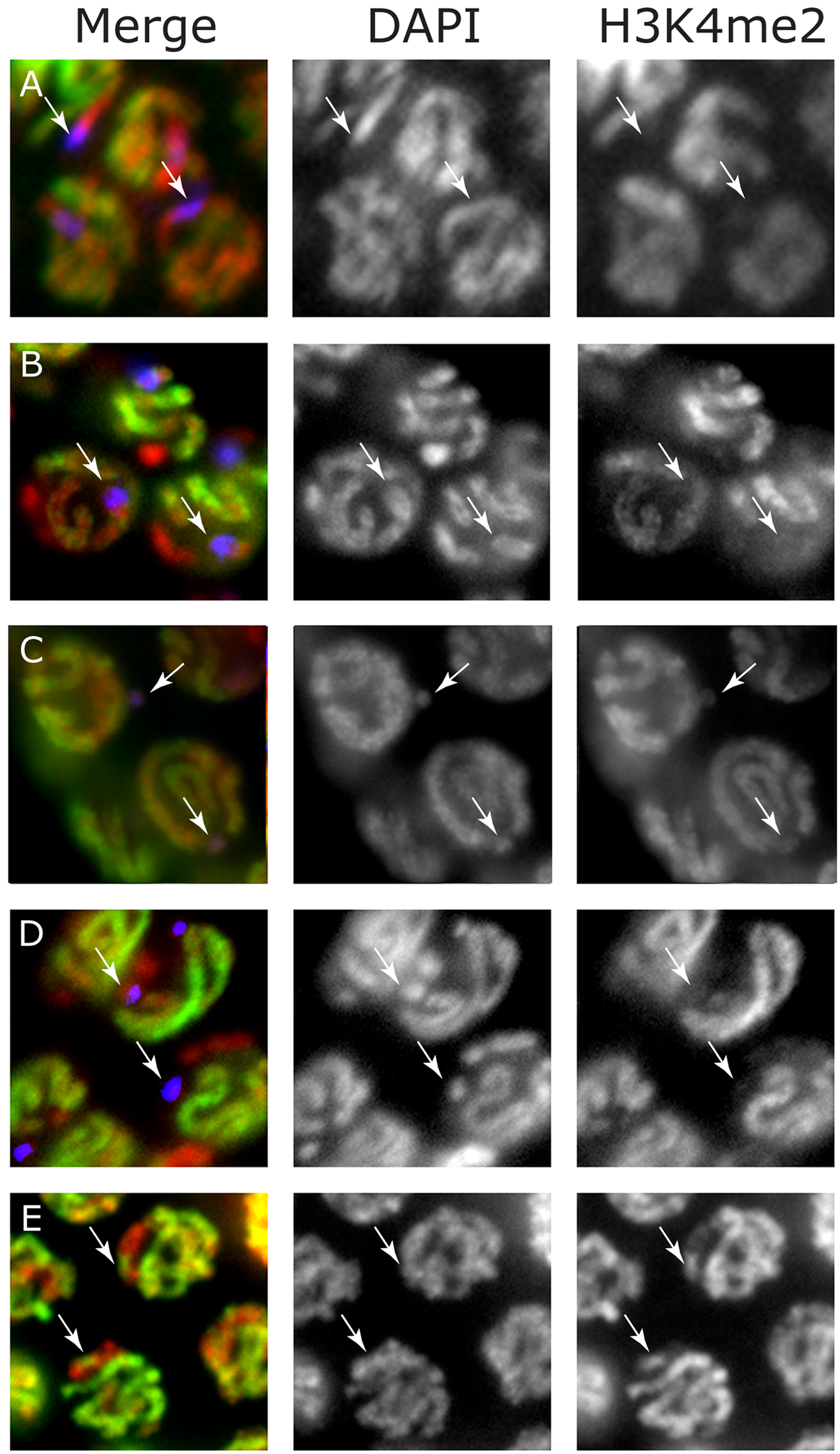

Supplement: Figure S2 — H3K4me2 in transgene chromatin in germ cells correlates with germline transcription. (A–E) Pachytene nuclei from adult hermaphrodites with DAPI (red), antibody against H3K4me2 (green), and transgene DNA FISH (blue). (A) X-linked, germline silent pes-10::GFP transgene (arrows) lacks H3K4me2, as does the rest of the X chromosome, in pachytene nuclei. (B) LG V-linked, germline silent mIs10 transgene (arrows) lacks H3K4me2 in pachytene nuclei. (C) Germline expressing Ex1336 extrachromosomal transgene (arrows) accumulates H3K4me2 in pachytene nuclei. (D) Germline silent Ex1336 extrachromosomal transgene (arrow) in wild-type background lacks H3K4me2 in pachytene nuclei. (E) X-linked, germline expressing his-24::GFP transgene accumulates H3K4me2 on X in pachytene nuclei (arrow) (FISH not shown). (3.49 MB TIF) [file pgen.1001391.s002.tif]

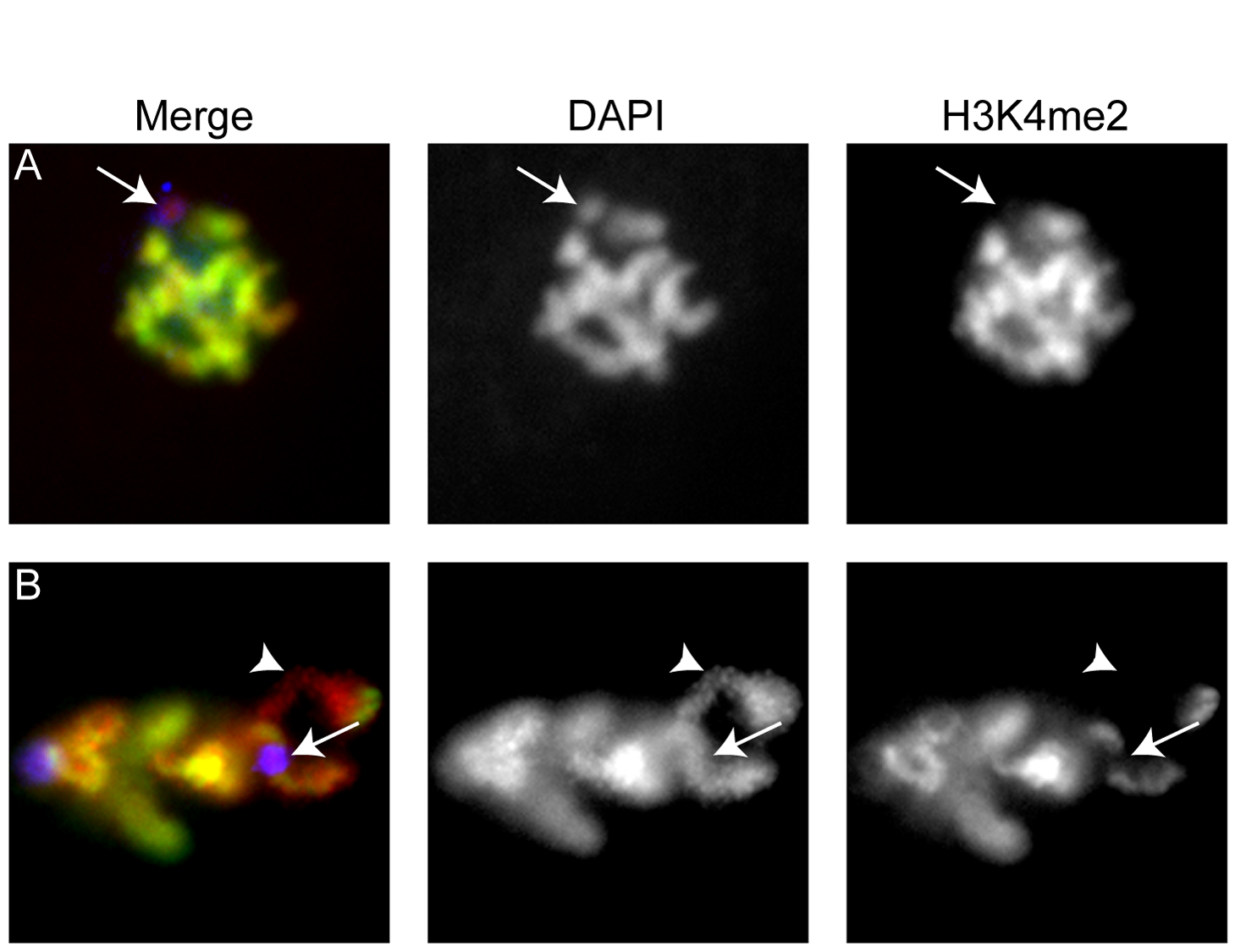

Supplement: Figure S3 — Germline repressed transgenes maintain imprinted chromatin in the early embryo. (A) Extrachromosomal array PD7271 (arrow) lacks H3K4me2 in embryo, as does the germline silent transgene ccIn3861 (B). (0.64 MB TIF) [file pgen.1001391.s003.tif]

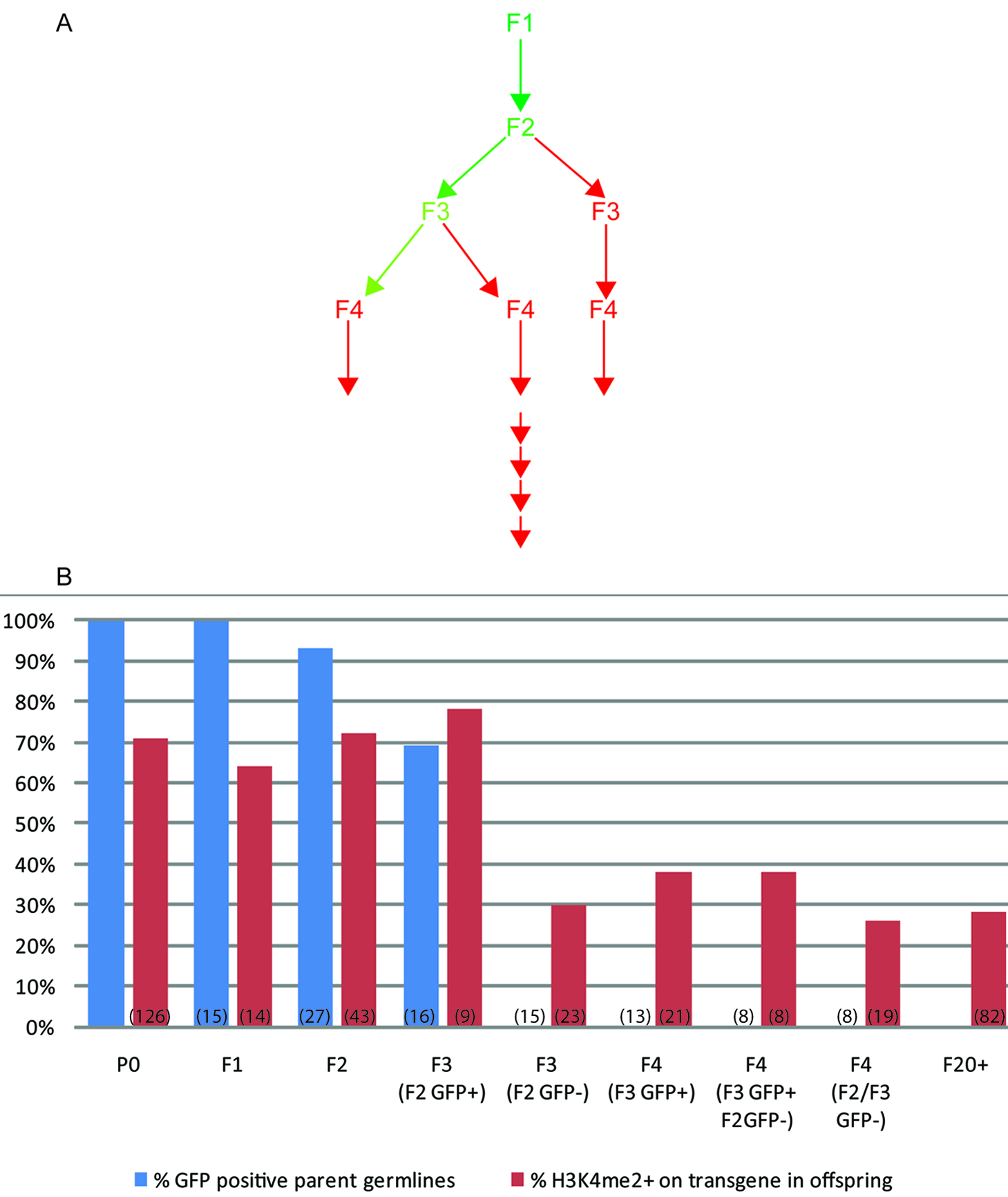

Supplement: Figure S4 — Transgene germline GFP expression versus transgene H3K4me2 in embryos. (A) Schematic of selection of animals during this experiment. At each generation animals were cloned out and allowed to lay embryos. Half of the animals were scored live for germline GFP expression and the remaining animals were dissected and their embryos analyzed by DNA FISH and anti-H3K4me2 immunofluorescence (B) % of animals LET-858::GFP fluorescence in the germline as adults at each generation (blue bars) vs. % of offspring from each generation retaining H3K4me2 on the transgene as embryos (red bars). Offspring from germline expressing parents and offspring from germline silenced parents were examined separately in the F3 and F4 generations as indicated. Number of animals scored is indicated in parentheses. (1.00 MB TIF) [file pgen.1001391.s004.tif]

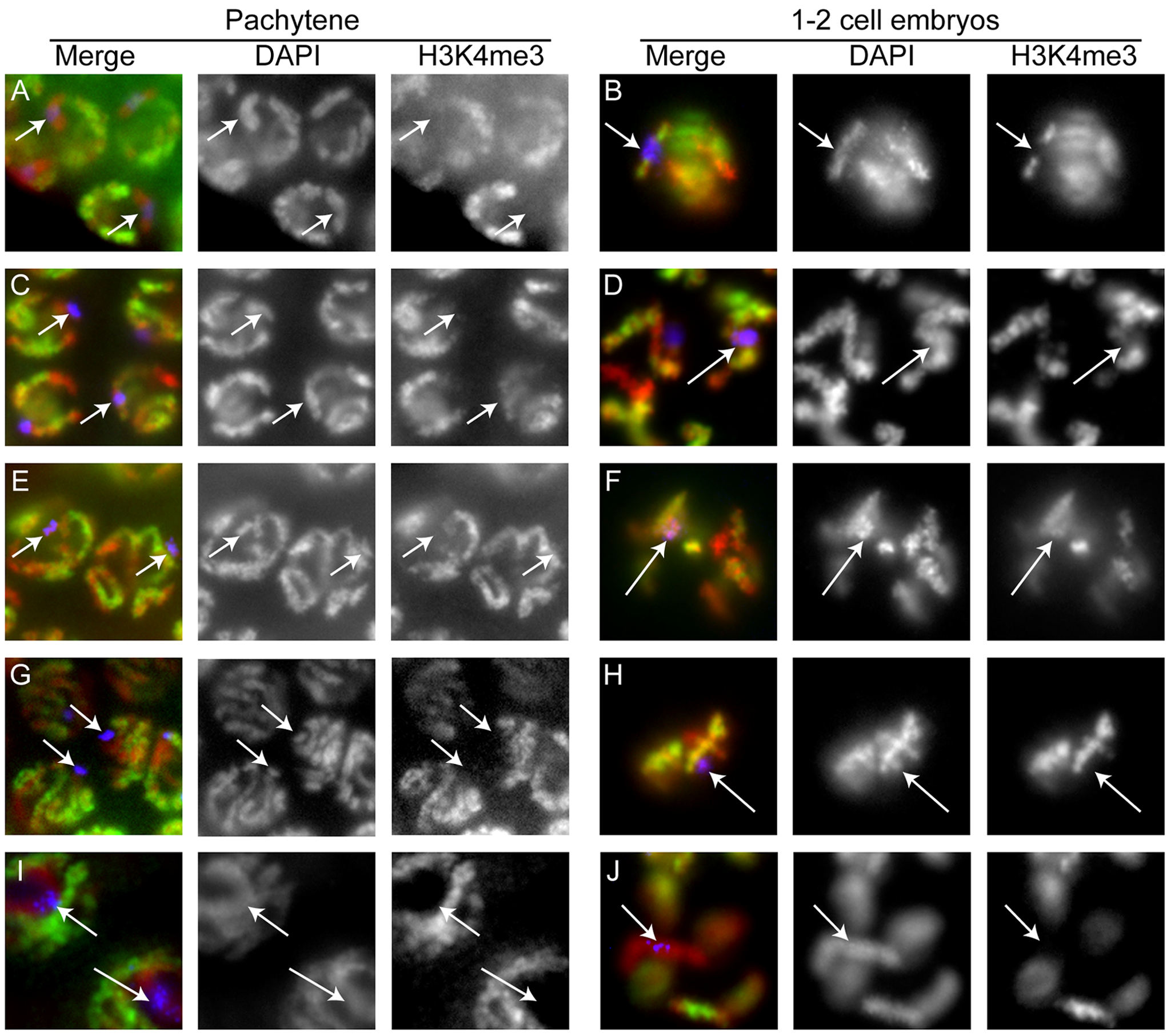

Supplement: Figure S5 — H3K4me3 of transgenes in germ cells and early embryo correlates with germline transcription. (A–J) Pachytene nuclei from adult hermaphrodites or one to two cell embryos with DAPI (red), antibody against H3K4me2 (green), and DNA FISH (blue). (A) X-linked, germline silent pes-10::GFP transgene lacks H3K4me3, as does the rest of the X chromosome, in pachytene nuclei. (B) X- linked pes-10::GFP transgene also lacks H3K4me3 on the Xm in a two cell embryo. (C) LG V-linked, germline silent mIs10 transgene (arrow) lacks H3K4me3 in pachytene nuclei. (D) LG V-linked, germline silent mIs10 transgene (arrow) also lacks H3K4me3 in a one cell embryo. (E) Germline expressing Ex1336 extrachromosomal transgene (arrows) does not appear to have H3K4me3 in pachytene nuclei. (F) Germline expressing Ex1336 extrachromosomal transgene (arrows) does accumulate H3K4me3 in a one cell embryo. (G) Germline silent Ex1336 extrachromosomal transgene (arrow) in wild-type background lacks H3K4me3 in pachytene nuclei. (H) Germline silent Ex1336 extrachromosomal transgene (arrow) in wild-type background lacks H3K4me3 in a one cell embryo. (I) X-linked, germline silent his-24::GFP transgene lacks H3K4me3 on X in pachytene nuclei (arrow). (J) X-linked germline silent his-24::GFP transgene lacks H3K4me3 on Xp in one cell embryo. (2.77 MB TIF) [file pgen.1001391.s005.tif]

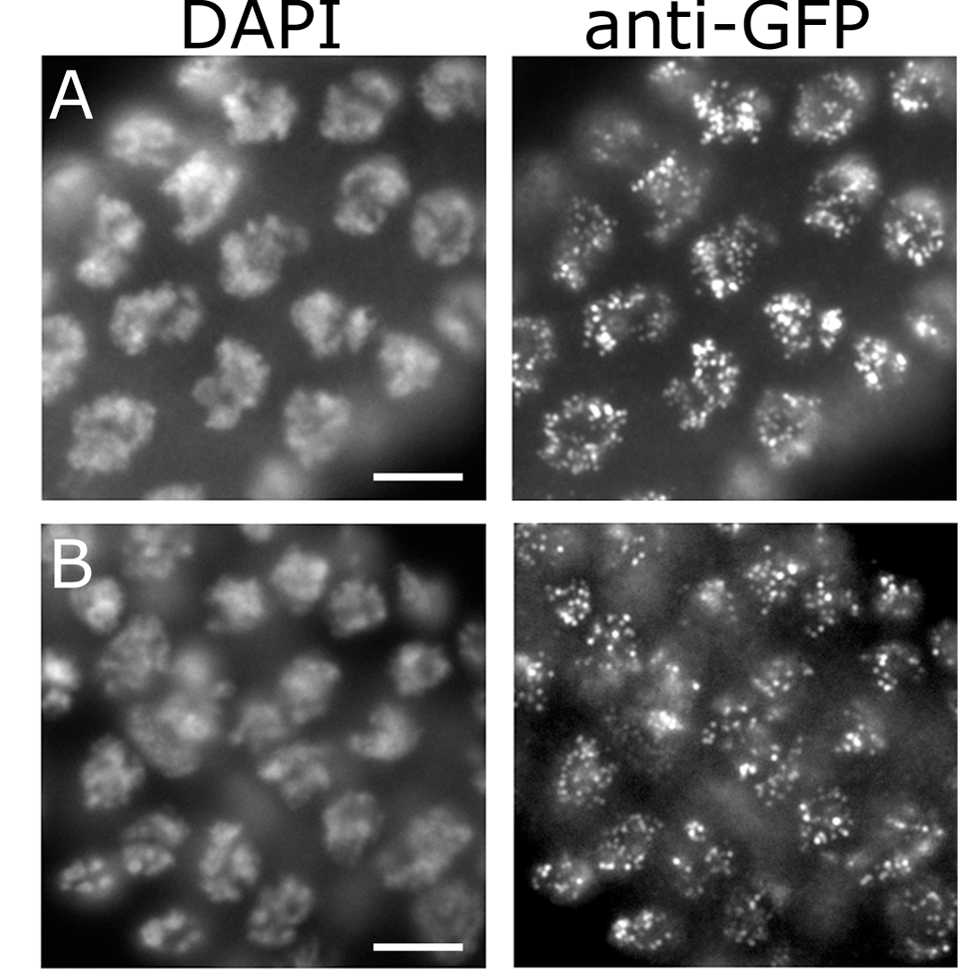

Supplement: Figure S6 — X-linked HIS-24::GFP transgene is expressed in the germline of both sexes. X-linked HIS-24::GFP is expressed in both hermaphrodite (A) and male (B) germ cells, as shown by antibody staining for GFP. Scale bars, 5 um. (0.77 MB TIF) [file pgen.1001391.s006.tif]

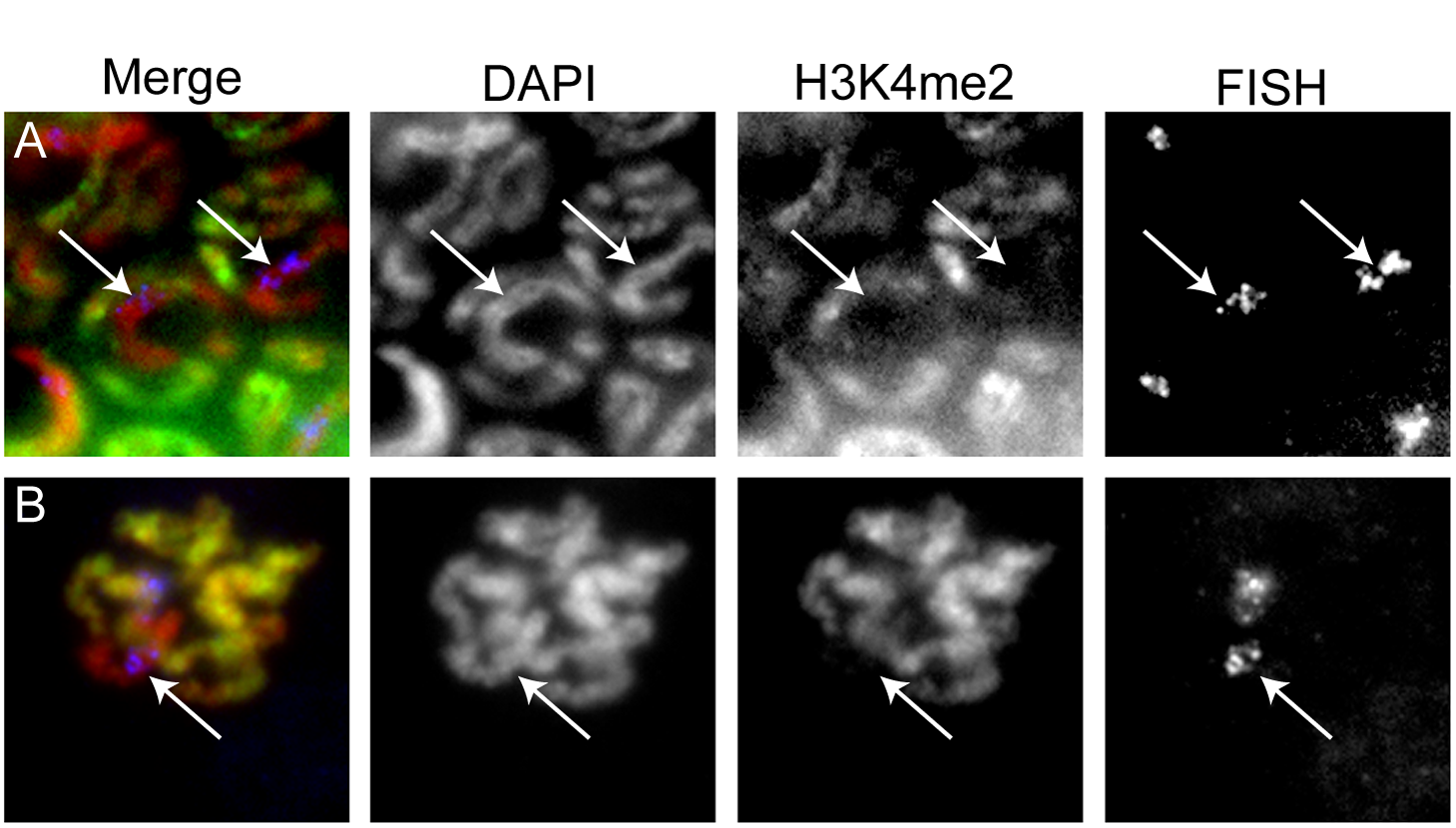

Supplement: Figure S7 — H3K4me2 is absent from Xp in pachytene and embryos in germline silent his-24::GFP. DAPI (red), antibody against H3K4me2 (green), and DNA FISH (blue). (A) X-linked his-24::GFP transgene (arrow) in a pachytene nuclei lacks H3K4me2 when transgene is germline silent. (B) X-linked his-24::GFP transgene (arrow) in a two cell embryo lacks H3K4me2 when transgene is germline silent. (0.81 MB TIF) [file pgen.1001391.s007.tif]

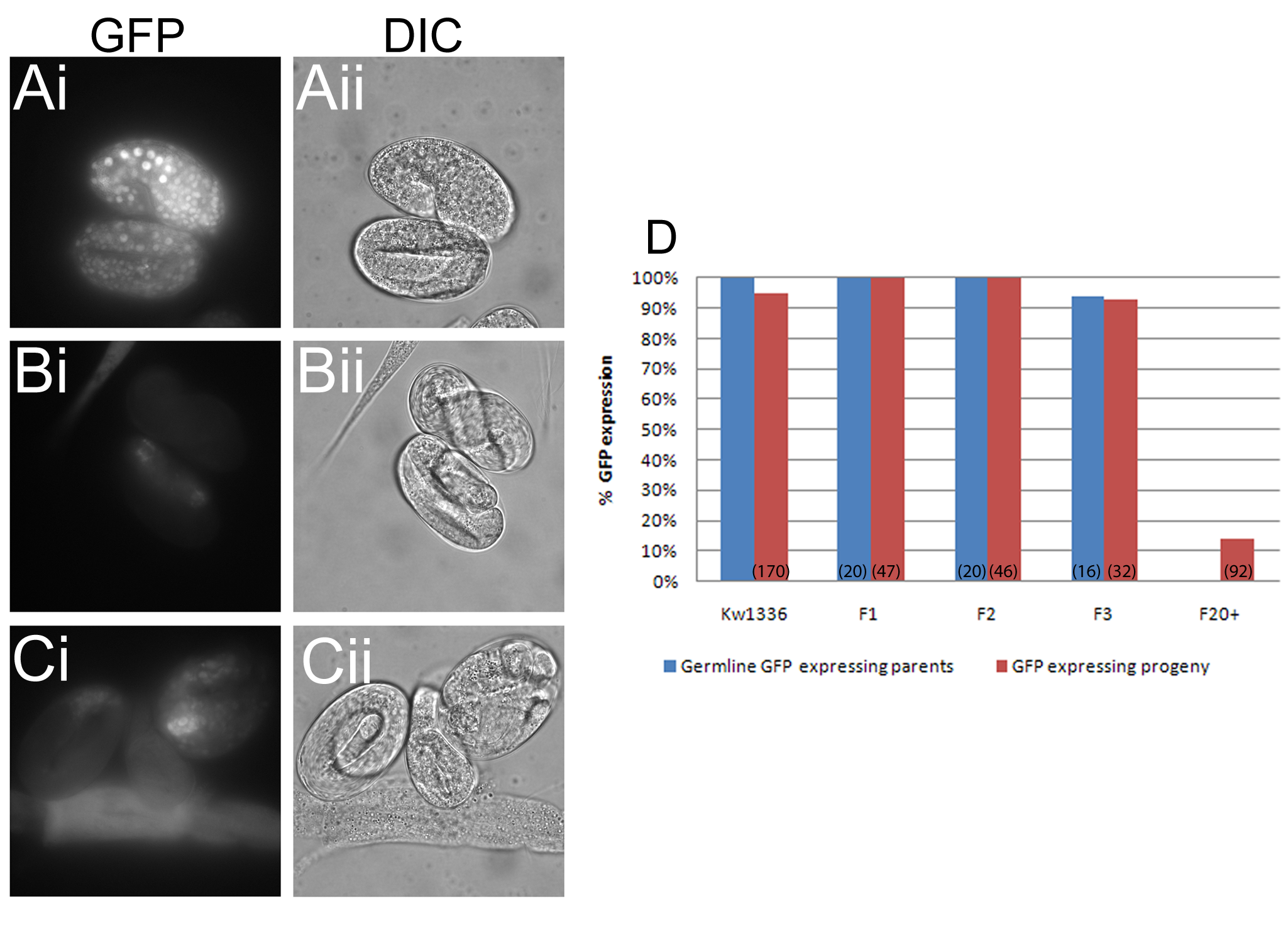

Supplement: Figure S8 — Transgene from germline silenced parents show reduced embryonic expression. GFP fluorescence (Ai–Ci) or DIC (Aii–Cii) microscopy of 1.5-fold to 3–fold stage live embryos. (Ai) 93% (n = 27) of embryos from KW1336 offspring from parents which express let-858:gfp in the germline show robust GFP expression in all nuclei. (Bi) 87% (n = 47) of offspring from outcrossed animals where germline expression of let-858:gfp was lost lacked any GFP positive nuclei (approximately 40% of offspring inherit the array). (Ci) Rare (13% n = 47) embryos from outcrossed parents where germline expression of let-858:gfp was lost with GFP positive nuclei (far right) have weaker and more variegated GFP expression than offspring from germline expressing parents (Ai). (D) % of animals expressing let-858::GFP in the germline as adults at each generation (blue bars) vs. % of offspring expressing somatic GFP as embryos (red bars). Number of animals scored is indicated in parentheses. (1.41 MB TIF) [file pgen.1001391.s008.tif]

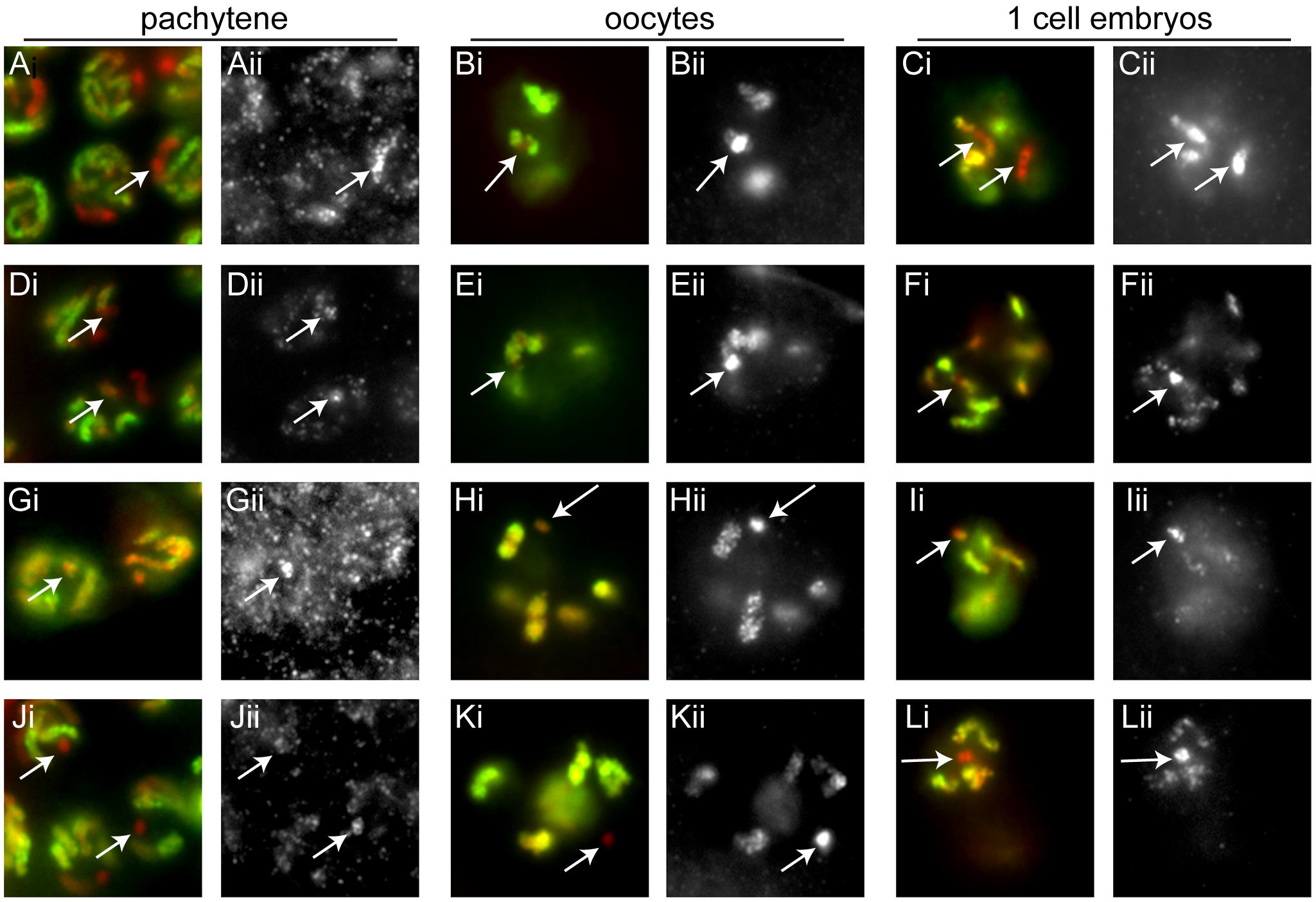

Supplement: Figure S9 — Transgenes are enriched for H3K9me3 in pachytene, oocytes, and early embryos independent of activity in the germline. Pachytene nuclei, oocytes, and nuclei from one or two cell embryos with H3K4me2 (green) and DAPI (red) (Ai–Li) or H3K9me3 (Aii–Lii) are shown. (Ai–Ci) X-linked, germline silent pes-10::GFP transgene (arrow) is enriched for H3K9me3 over autosomes and surrounding X-chromatin(Aii–Cii), (Di–Fi) LG V-linked, germline silent mIs10 transgene identified by lack H3K4me2 (arrow) is enriched for H3K9me3 over autosomes and surrounding chromatin (Dii–Fii). (Gi–Ii) Germline expressing Ex1336 extrachromosomal transgene (arrows) is enriched for H3K9me3 over autosomes, particularly in oocytes and early embryos (Gii–Iii). (Ji–Li) Germline silent extrachromosomal array PD7271 (arrow) is enriched for H3K9me3 over autosomes, particularly in oocytes and early embryos (Jii–Lii). (1.74 MB TIF) [file pgen.1001391.s009.tif]

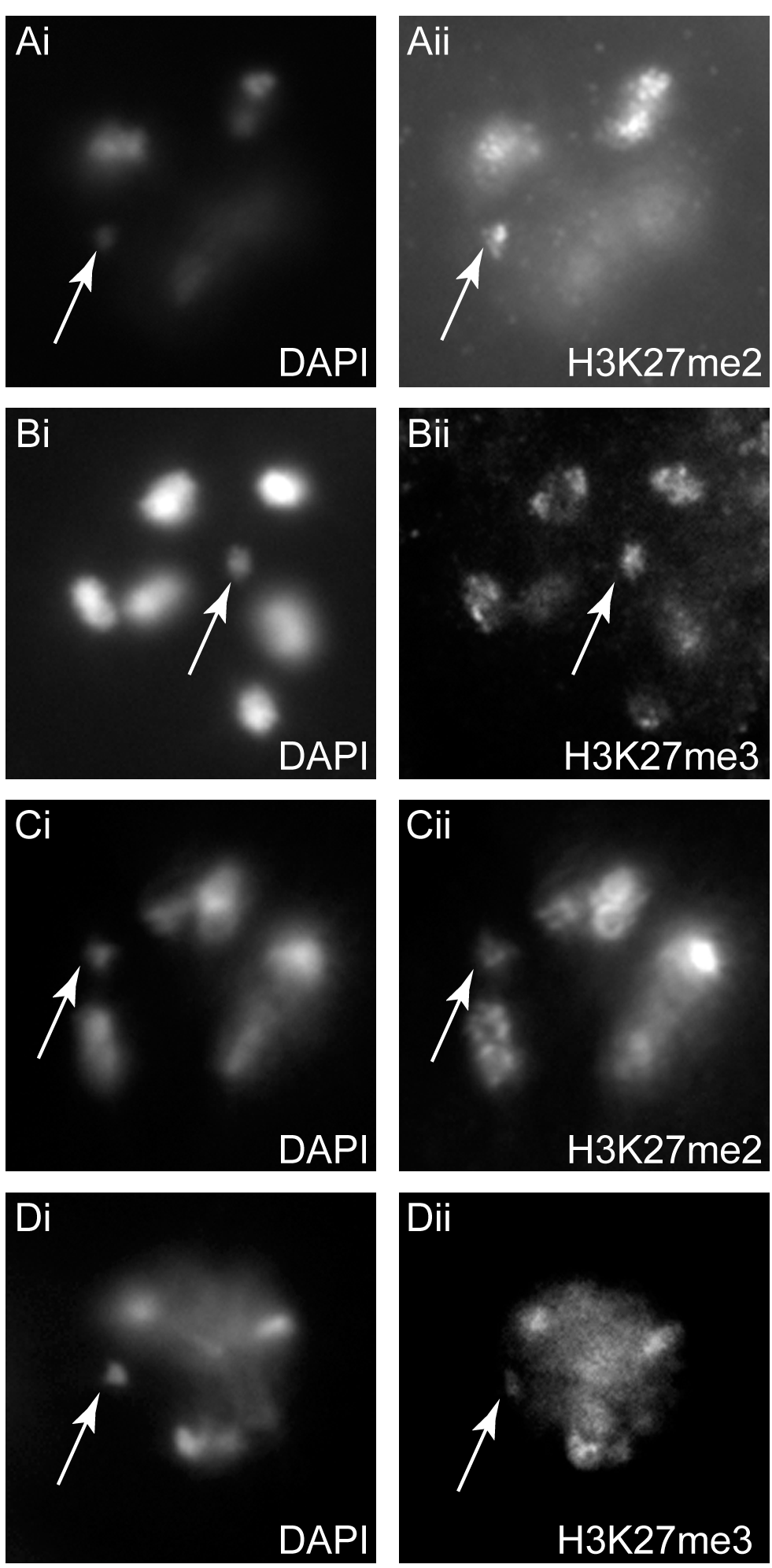

Supplement: Figure S10 — H3K27me2 and H3K27me3 in transgene chromatin do not noticeably correlate with transgene expression. Oocytes with DAPI (Ai–Di) and H3K27me2 (Aii and Cii) or H3K27me3 (Bii and Dii) are shown. (Ai–Bi) Extrachromosomal germline silent transgene PD7271 (arrow) is decorated with H3K27me2 (Aii) and H3K27me3 (Bii) at levels similar to autosomes. (Ci–Di) Extrachromosomal germline expressing transgene KW1336 (arrow) is decorated with H3K27me2 (Cii) and H3K27me3 (Dii) at levels similar to autosomes. The PD7271 transgene array is repetitive and silenced; the KW1336 array is more complex and expresses in germ cells. Neither of these characteristics correlate with presence or absence of H3K27me2/3 as levels on both arrays are similar to levels observed on chromosomes in the same nuclei. (0.81 MB TIF) [file pgen.1001391.s010.tif]
